# Adeno-associated virus–based gene therapy treats inflammatory kidney disease in mice

**DOI:** 10.1172/JCI174722

**Published:** 2024-08-15

**Authors:** Guochao Wu, Shuya Liu, Julia Hagenstein, Malik Alawi, Felicitas E. Hengel, Melanie Schaper, Nuray Akyüz, Zhouning Liao, Nicola Wanner, Nicola M. Tomas, Antonio Virgilio Failla, Judith Dierlamm, Jakob Körbelin, Shun Lu, Tobias B. Huber

**Affiliations:** 1 III. Department of Medicine,; 2 Hamburg Center for Kidney Health (HCKH),; 3 Bioinformatics Core,; 4 Department of Oncology, Hematology and Bone Marrow Transplantation with Section Pneumology, and; 5 Microscopy Imaging Facility, University Medical Center Hamburg-Eppendorf, Hamburg, Germany.

**Keywords:** Nephrology, Therapeutics, Autoimmune diseases, Endothelial cells, Gene therapy

## Abstract

Adeno-associated virus (AAV) is a promising in vivo gene delivery platform showing advantages in delivering therapeutic molecules to difficult or undruggable cells. However, natural AAV serotypes have insufficient transduction specificity and efficiency in kidney cells. Here, we developed an evolution-directed selection protocol for renal glomeruli and identified what we believe to be a new vector termed AAV2-GEC that specifically and efficiently targets the glomerular endothelial cells (GEC) after systemic administration and maintains robust GEC tropism in healthy and diseased rodents. AAV2-GEC–mediated delivery of IdeS, a bacterial antibody-cleaving proteinase, provided sustained clearance of kidney-bound antibodies and successfully treated antiglomerular basement membrane glomerulonephritis in mice. Taken together, this study showcases the potential of AAV as a gene delivery platform for challenging cell types. The development of AAV2-GEC and its successful application in the treatment of antibody-mediated kidney disease represents a significant step forward and opens up promising avenues for kidney medicine.

## Abstract 2

A kidney-specific adeno-associated virus–based gene therapy efficiently targets the glomerular endothelial cells and prevents the onset and progression of inflammatory kidney disease in mice.

## Introduction

Most kidney diseases are associated with the dysfunction of the glomerular filtration barrier (GFB), which comprises 3 layers, including the glomerular endothelial cells (GEC), the glomerular basement membrane (GBM) and podocytes (1). GEC are fenestrated endothelial cells (EC) covered by a negatively charged surface layer known as the glycocalyx. Podocytes are specialized epithelial cells with foot processes spanned by cell-cell junctions known as slit diaphragms. The GBM is formed by secreted products from both GEC and podocytes. The functionality of the GFB relies strictly on its structural integrity, and breakdown of the GFB leads to the loss of kidney filtration function (2). Current interventions for kidney disease mostly target complications or manifestations of the disease and have limited disease-modifying effects (3). In past years, multiple molecules with therapeutic potential for the GFB have been identified. To date, more than 80 gene mutations or variants have been found to cause GFB disorders (4), and an increasing number of clinical trials are ongoing for drugs targeting the GFB (5). Therefore, the GFB is an important target for novel kidney therapies.

GFB-targeting therapies not only involve rare diseases caused by genetic defects in the GEC or podocytes, but also common diseases characterized by glomerulosclerosis or glomerulonephritis, which are initiated in these 2 cell types. However, targeting the GFB is challenging (5). Traditional drugs like small molecules and monoclonal antibodies show limited efficacy or undesired side effects due to the lack of cell- or tissue-targeting specificity (5). Encouragingly, recent innovations in gene therapy made it possible to deliver therapeutic genetic cargo to difficult or previously undruggable cells, which can substantially improve the therapeutic efficacy.

Adeno-associated virus (AAV) is regarded as a promising viral-based platform for in vivo gene delivery (6), which has been licensed by the FDA and European Medicines Agency (EMA) and proven to have benefits in many genetic diseases, such as hemophilia and spinal muscular atrophy (7). AAV has been successfully applied in vivo as a delivery tool for the treatment of genetic diseases affecting many organs, but such applications are not yet available in the kidney (8). Natural AAV serotypes show insufficient targeting specificity and transduction efficiency in kidney cells and thus do not meet the requirements as a delivery tool for kidney-targeting therapy (9, 10). As the kidney is a complex organ comprising a variety of different cell types and tissues, approaches to broaden the tropism of AAV and screenings for kidney-specific AAV vectors are essential (11).

In this study, we aimed to discover new vectors targeting the renal glomerulus. We developed a kidney-specific selection protocol based on a previously described methodology (12) and screened a random AAV2 display peptide library in vivo. By integrating the experimental and bioinformatics workflows, we identified what we believe to be a new vector, termed AAV2-GEC, which specifically and efficiently targeted the GEC after systemic administration. AAV2-GEC exhibited robust GEC tropism in healthy C57BL/6J, Balb/c, BTBR mice, Sprague Dawley (SD) rats, and disease models that cause GEC damage. It also exhibited increased transduction efficiency and specificity in human primary GEC compared with WT AAV2 (AAV2-WT). Further, the potential of AAV2-GEC for kidney-targeting therapy was evaluated by delivering a bacterial cysteine proteinase, the IgG-degrading enzyme of Streptococcus pyogenes (IdeS), to the GEC. IdeS, also known as imlifidase, is a medication for the desensitization of highly sensitized patients undergoing kidney transplantation (13) and is currently being tested in clinical trials for therapeutic cleavage of kidney-bound IgG in patients with circulating anti-GBM antibodies (14). We show that AAV2-GEC-IdeS transduction efficiently produced IdeS in GEC, which provided sustained clearance of IgG and successfully prevented the onset and progression of anti-GBM glomerulonephritis in mice.

## Results

### In vivo selection of a random peptide library enriched glomeruli-targeted capsids.

**Figure 1:**
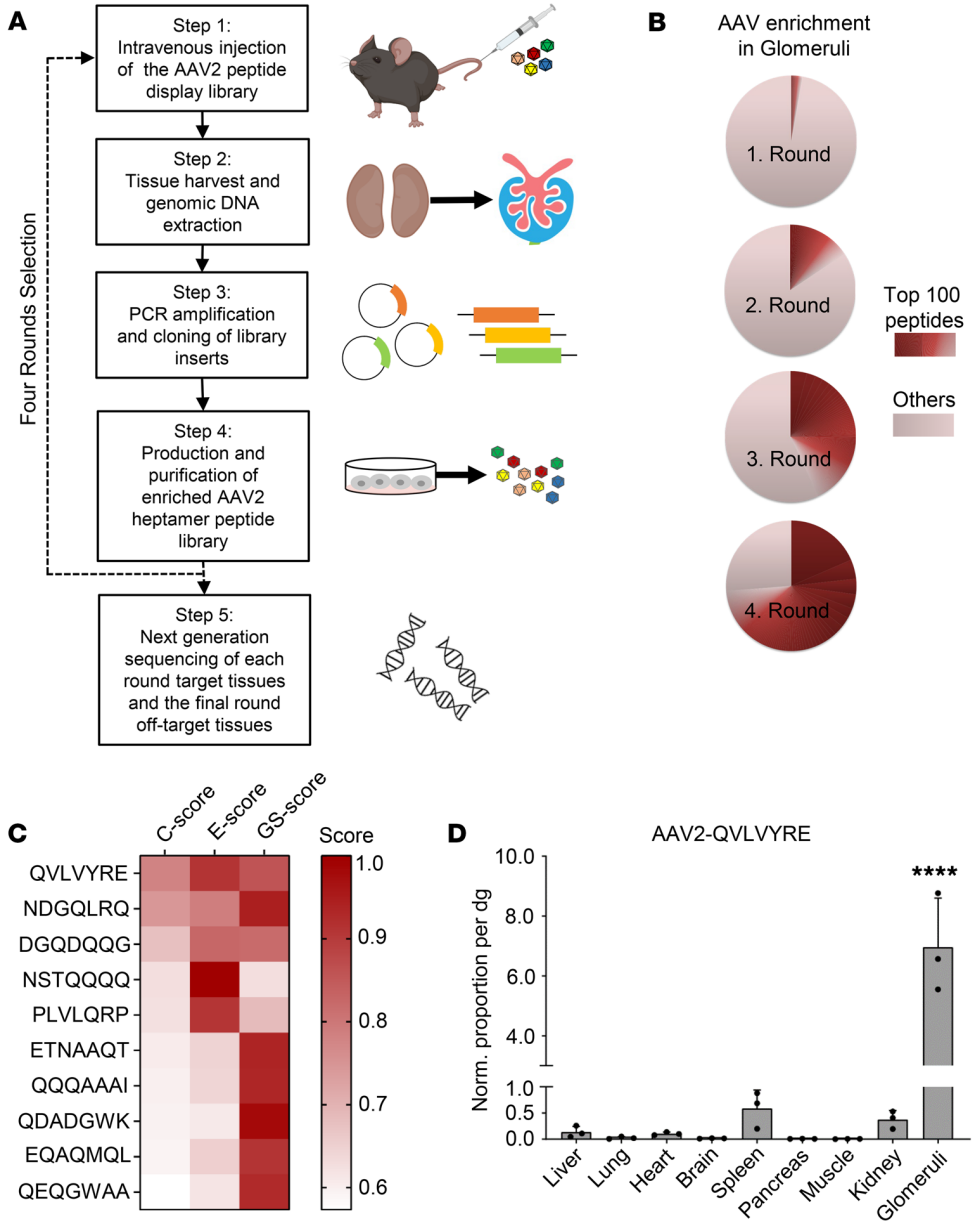
In vivo selection of the AAV2 peptide display library identified capsids enriched in renal glomeruli. (**A**) Schematic overview of in vivo selection in the murine kidney. (**B**) Pie charts demonstrating the distribution of peptide variants in each selection round. The frequency of particular peptide inserts was determined by NGS. “Others” indicates the occurrence of peptide variants ranked below the “top 100 peptides” in the total pool. (**C**) Heatmap demonstrating the top 10 peptide variants enriched in the glomerulus ranked by C scores. The combined C score (by multiplying GS and E) described the peptide performance regarding specificity (GS score) and efficacy (E score) with an ideal value of 1. (**D**) Quantification of vector genome distribution by qPCR. The number of vector genomes was quantified and normalized to vector copy numbers per diploid genome (vg/dg). Values are mean + SD. Significance was determined by 1-way ANOVA with Dunnett’s test, ****P < 0.0001 in all comparisons (glomeruli versus other organs).

To select kidney-specific AAV capsids, we used an AAV2-displayed random heptamer peptide library (15, 16) with a calculated plasmid diversity of 1.5 × 10^8^ (17) and we established an in vivo screening protocol for kidneys based on a previous report (12) (Figure 1A).

Since the kidney is a complex organ in terms of both anatomical structure and a large number of different cell types, it is important to monitor the selection kinetics and adjust the selection pressure during the process of in vivo screening. In the first 2 rounds of selection, the AAV library fragments were rescued from the genomic DNA of the whole kidney. From the third round of selection, we increased the selection pressure by rescuing the genomic DNA only from the isolated glomeruli instead of the whole kidney. Next generation sequencing (NGS) was performed after each round of selection to thoroughly analyze the enriched peptides from the target organ. After the fourth round of selection, we detected a dramatic enrichment. As expected, the number of peptide sequences decreased in each round of selection, whereas the percentage of the top 100 enriched peptides increased accordingly (Figure 1B and Table 1). The sequences of the top 10 enriched peptides in each selection round are listed in Table 2.

In the last round of selection, NGS was performed to analyze the peptides from off-target organs. We evaluated all enriched peptides by a rating system based on NGS data that reflects the relative frequency of a given peptide in the target tissue and its distribution in the target tissue compared with the off-target organs (see Methods) (16). In this study, the enrichment score (E score) reflected the changes in relative abundance from the third to fourth round of selection in glomeruli. The general tissue specificity score (GS score) reflected the relative abundance in glomeruli compared with multiple off-target organs. The combined score (C score) was determined by multiplying GS and E, reflecting the performance of a given peptide regarding both targeting specificity and efficacy. Thus, the enriched peptides were ranked by C scores. In the top 10 peptides (Figure 1C), QVLVYRE didn’t have the highest E score, but it outperformed other sequences with a better GS score, suggesting that it was not only highly abundant but also highly specific in glomeruli (Figure 1C). We further evaluated the targeting specificity and efficacy of AAV2-QVLVYRE by quantifying the distribution of the vector genome across all major organs. Quantitative PCR (qPCR) showed that AAV2-QVLVYRE was at least 10-fold more dominant in glomeruli compared with other organs, including the whole kidney (Figure 1D). Taken together, QVLVYRE was chosen as the most promising peptide targeting the renal glomerulus.

### AAV2 vector displaying the QVLVYRE peptide specifically transduced the GEC.

To evaluate the *in vivo* transduction profile of AAV2-QVLVYRE, we generated a self-complementary AAV reporter vector carrying the *GFP* gene driven by the constitutive CMV promoter and intravenously injected adult C57BL/6J mice with a dose of 5E12 vg/kg. Transgene expression was analyzed in different organs 2 weeks after the injection.

**Figure 2:**
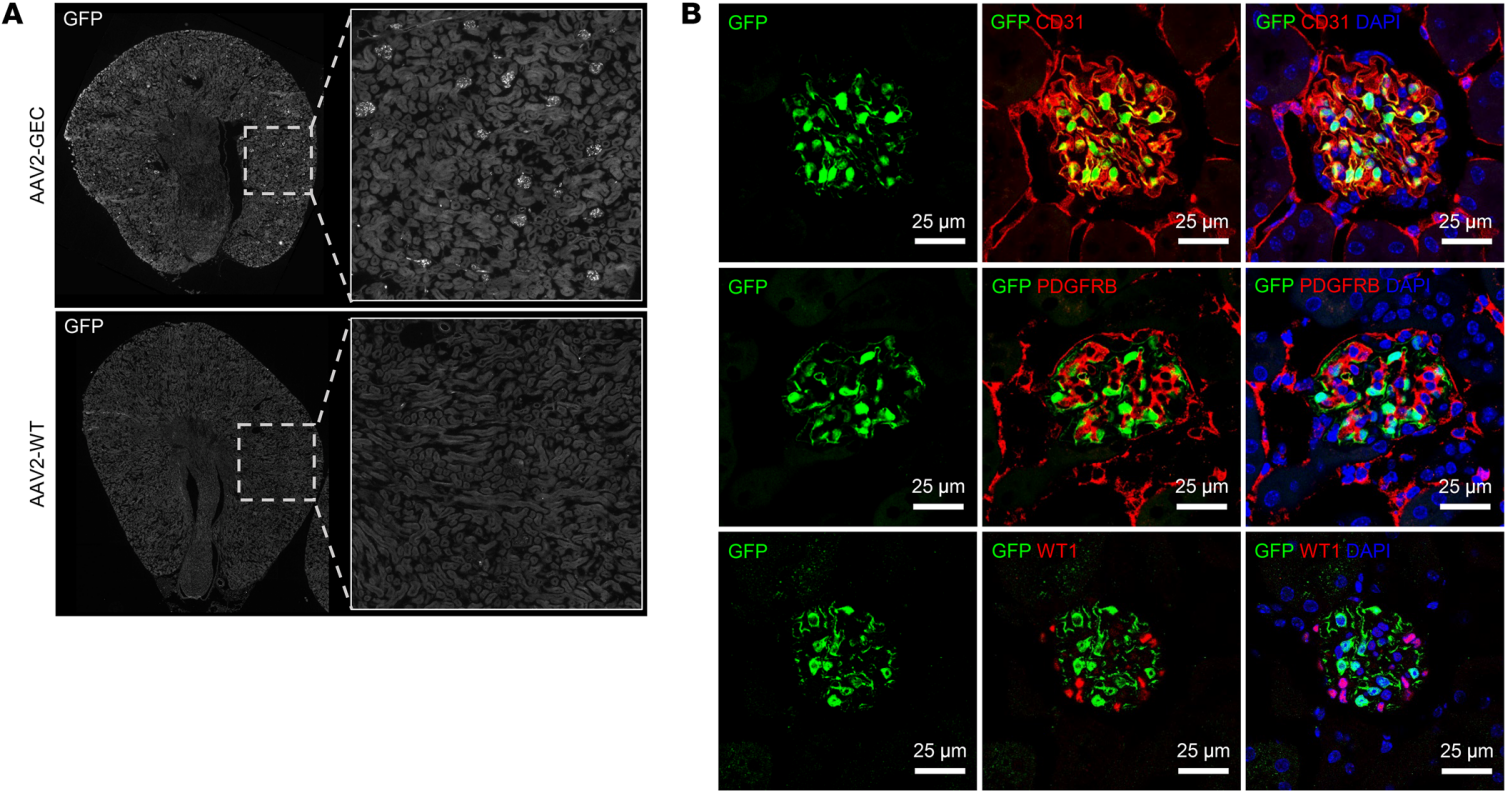
AAV2-GEC specifically transduced the GEC. (**A**) Representative overview images of AAV2-GEC and AAV2-WT mediated GFP expression in kidneys from C57BL/6J. Original magnification x10. (**B**) AAV2-GEC mediated GFP expression was detected in the GEC marked by anti-CD31 antibody. Mesangial cells were marked by anti-PDGFRB antibody. Podocytes were marked by anti-WT1 antibody. Nuclei were counterstained with DAPI. Scale bars: 25 μm.

In the kidney, immunofluorescent staining (IF) showed that the GFP expression mediated by AAV2-QVLVYRE was restricted to the glomerulus and revealed excellent transduction efficiency in all renal glomeruli (Figure 2A). We further confirmed that AAV2-QVLVYRE specifically transduced the GEC, which was marked by CD31, but not podocytes marked by Wilms tumor protein (WT1), or mesangial cells marked by platelet-derived growth factor receptor β (PDGFRB) (Figure 2B). AAV2-QVLVYRE was hence termed AAV2-GEC.

The transduction properties of AAV2-GEC were compared with its parental AAV2-WT. No GFP expression was detected in the kidney of AAV2-WT–injected mice (Figure 2A). GFP expression was strong in the liver and heart and moderate in the spleen of AAV2-WT–injected mice, whereas it was far weaker in all of the same organs of AAV2-GEC–injected mice (Supplemental Figure 1A; supplemental material available online with this article; https://doi.org/10.1172/JCI174722DS1). Notably, GFP-positive cells were not colocalized with endothelial cell markers in the liver, heart, and spleen of AAV2-GEC–injected mice (Supplemental Figure 1B). AAV2-GEC–mediated GFP expression was analyzed over 360 days after intravenous injection (Supplemental Figure 2A) and the GFP signal intensity was quantified (Supplemental Figure 2B). Throughout the whole period, glomerular GFP peaked at day 14, was stable at high levels until day 120, and decreased from day 240. GFP signal was dominant in glomeruli, but from day 120 it was also detected in some EC in the tubular segment. Additionally, liver and spleen histology was analyzed (Supplemental Figure 3). No obvious histological lesions were observed over 360 days, indicating no tissue toxicity due to the injection of AAV2-GEC. Taken together, AAV2-GEC mediates specific and prolonged GFP expression in the GEC for at least 120 days upon intravenous injection.

### AAV2-GEC maintained robust tropism in GFB-damaged mice.

GEC are highly differentiated EC characterized by their unique fenestrae and surface layer glycocalyx, which are essential for glomerular filtration (18). The differentiation and permeability of GEC are also regulated by podocytes (5). Under disease conditions, the breakdown of the GFB due to the injury of GEC or podocytes could lead to changes in the GEC-directed tropism of our targeted AAV2. Therefore, we evaluated the AAV2-GEC tropism in mouse models with damaged GFB.

**Figure 3:**
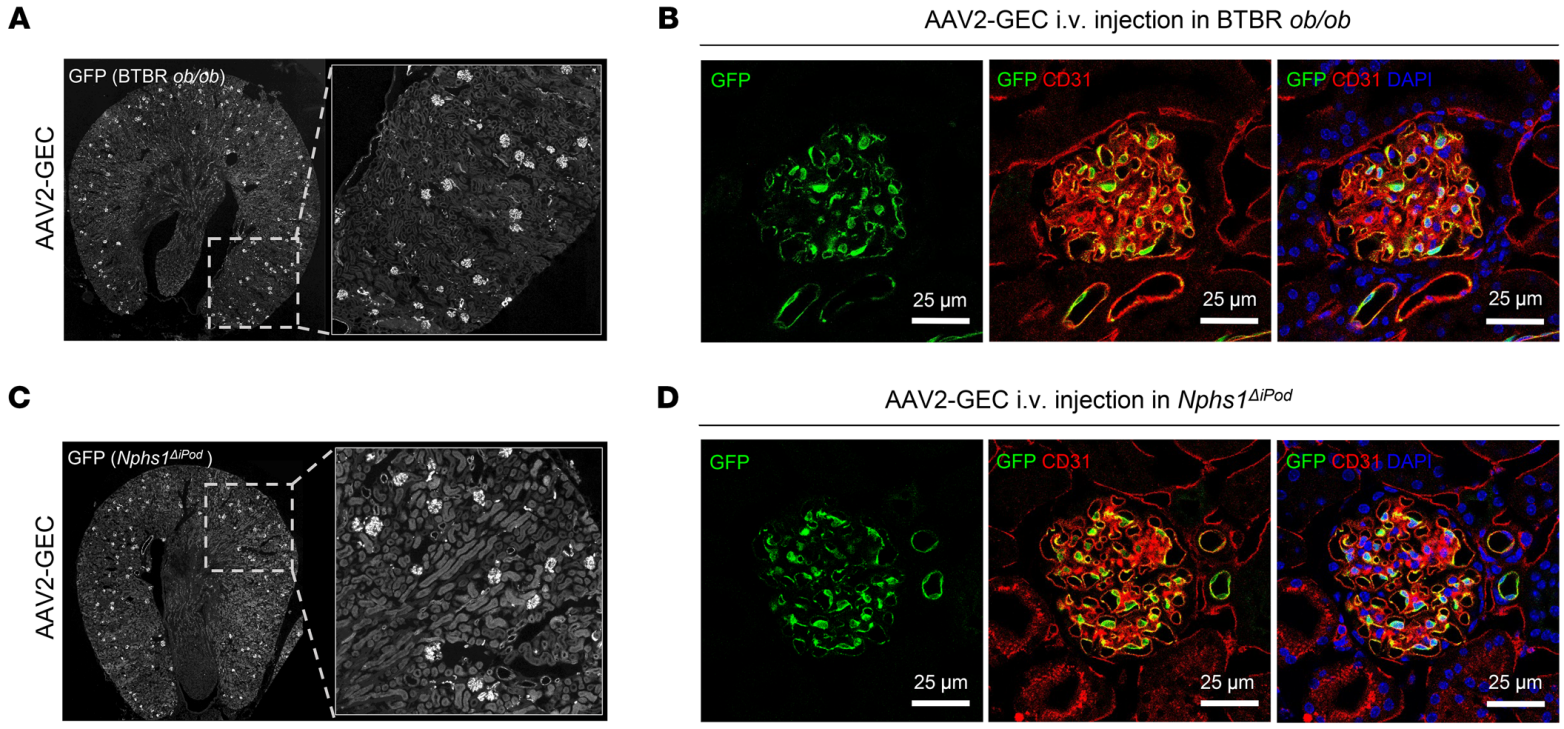
AAV2-GEC maintained robust tropism in BTBR^ob/ob^ and Nphs1^ΔiPod^ mice. (**A**) Representative overview images of AAV2-GEC–mediated GFP expression in kidneys from BTBR *ob/ob* mice. Original magnification x10. (**B**) GFP expression was detected in the GEC marked by anti-CD31 antibody. (**C**) Representative overview images of AAV2-GEC mediated GFP expression in kidneys from *Nphs1*^ΔiPod^ mice. Original magnification x10. (**D**) GFP expression was detected in the GEC marked by anti-CD31 antibody. Nuclei were counterstained with DAPI. Scale bars (**B** and **D**): 25 μm.

GEC injuries such as the loss of fenestrae and glycocalyx disruption are typically induced by hyperglycemia in diabetic kidney disease (DKD) (18). Previous studies report that BTBR mice expressing homozygous spontaneous obesity mutations (BTBR*^ob/ob^*), a well-characterized DKD mouse model, exhibits early onset of hyperglycemia (19) and shows significant fenestrae changes in GEC (20). We thus used BTBR*^ob/ob^* to evaluate the transduction profile of AAV2-GEC under the GEC injury condition. AAV2-GEC-GFP was intravenously injected in 16–18 week-old BTBR*^ob/ob^* mice with a dose of 5 × 10^12^ vg/kg. Two weeks after injection, IF of kidney sections showed robust and efficient GFP expression in GEC (Figure 3, A and B) but not in podocytes or mesangial cells (Supplemental Figure 4A).

In podocytes, Nephrin is one of the essential slit diaphragm proteins. Loss of Nephrin at adult age results in podocyte injury and GFB leakage (21). We evaluated the transduction profile of AAV2-GEC in *Nphs1*^ΔiPod^ mice, which have induced Nephrin-deficiency in podocytes after doxycycline administration (21). AAV2-GEC-GFP was intravenously injected in *Nphs1*^ΔiPod^ mice 12 weeks after knock-out induction with a dose of 5 × 10^12^ vg/kg. IF was performed on the kidney sections after 2 weeks, showing robust and efficient GFP expression in GEC (Figure 3, C and D) but not in podocytes or mesangial cells (Supplemental Figure 4B).

To compare the transduction efficiency of AAV2-GEC in healthy and diseased states, AAV2-GEC-GFP was injected in C57BL/6J, BTBR*^ob/ob^*, BTBR WT, *Nphs1*^ΔiPod^ and noninduced *Nphs1*^ΔiPod^ mice (hereafter referred to as *Nphs1^ctrl^*). GEC were isolated for transcriptome analysis. There were no significant differences in *GFP* expression in BTBR*^ob/ob^*, BTBR WT, *Nphs1*^ΔiPod^, and *Nphs1^ctrl^* compared with C57BL/6J mice, and also no significant differences between BTBR WT and BTBR*^ob/ob^* and between *Nphs1*^ΔiPod^ and *Nphs1^ctrl^* mice (Supplemental Figure 5A). To investigate the effect of AAV transduction on GEC function, the gene expression levels in AAV transduced versus nontransduced GEC were compared. We identified 68 differentially expressed genes in AAV-transduced versus nontransduced cells (DEGs) (21 up and 47 downregulated) in BTBR*^ob/ob^* mice and 24 DEGs (20 up and 4 downregulated) in *Nphs1*^ΔiPod^ mice, whereas no significant DEGs were identified in C57BL/6J mice (Supplemental Table 1). The most significant DEGs are shown in a heatmap (Supplemental Figure 5B). Gene set enrichment analysis (GSEA) predicted significant gene ontology (GO) in AAV transduced GEC in BTBR*^ob/ob^* and *Nphs1*^ΔiPod^ mice (Supplemental Table 1), showing that RNA binding and ribosome biogenesis were affected by AAV transduction or transgene (GFP) expression (Supplemental Figure 5C).

### AAV2-GEC maintained robust tropism in Balb/c mice and SD rats.

**Figure 4:**
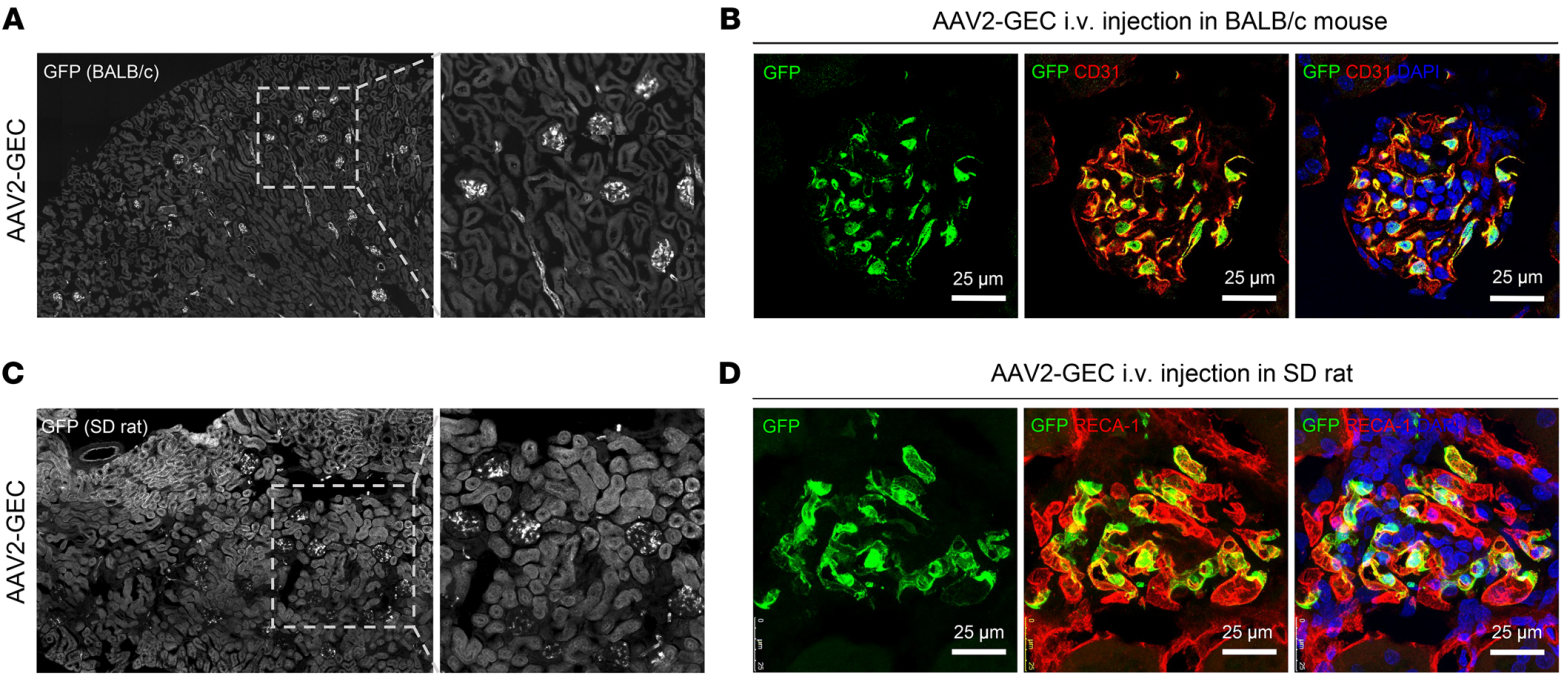
AAV2-GEC maintained robust tropism in Balb/c mice and SD rats. (**A**) Representative overview images of AAV2-GEC mediated GFP expression in kidneys from Balb/c mice. (**B**) GFP expression was detected in the GEC marked by anti-CD31 antibody. (**C**) Representative overview images of AAV2-GEC mediated GFP expression in kidneys from SD rats. (**D**) GFP expression was detected in the GEC marked by anti–RECA-1 antibody. Nuclei were counterstained with DAPI. Scale bars (**B** and **D**): 25 μm. Original magnification: x20 (**A**) and x10 (**C**).

Since the transduction by AAV vectors may vary substantially between strains and species (22, 23), we evaluated AAV2-GEC tropism in adult Balb/c mice and SD rats. AAV2-GEC-GFP was intravenously injected in Balb/c mice with a dose of 5 × 10^12^ vg/kg. After 2 weeks, IF was performed on the kidney sections. We observed robust and efficient GFP expression in the GEC of the Balb/c mice (Figure 4, A and B). Similar tropism of AAV2-GEC was also observed in the SD rat at the dose of 5 × 10^12^ vg/kg, in which GEC were marked by endothelial cell–specific biomarker rat endothelial cell antigen 1 (RECA-1) (Figure 4, C and D). Of note is that the dose of 5 × 10^12^ vg/kg used here was below the low dose range (1.2 × 10^13^ vg/kg) for rats, as previously reported (24).

### AAV2-GEC exhibited enhanced transduction in human primary GEC.

To validate the targeting efficacy of AAV2-GEC on human primary GEC, we applied AAV2-GEC or AAV2-WT luciferase reporter vectors. Reporter gene activity of AAV2-GEC was 2-fold higher than that of AAV2-WT (Supplemental Figure 6A). To evaluate cell type specificity of AAV2-GEC, transduction efficiency was also investigated in other human glomerular cell types, including immortalized podocytes and human primary mesangial cells. AAV2-GEC showed a 95% decrease in reporter gene activities compared with AAV2-WT in both glomerular cell types (Supplemental Figure 6, B and C). These data suggest that AAV2-GEC exhibited enhanced transduction in human GEC compared with AAV2-WT, but not in other human glomerular cell types.

### AAV2-GEC delivery of IdeS successfully treated anti-GBM glomerulonephritis.

To investigate the feasibility of using AAV2-GEC for in vivo delivery of therapeutic transgenes, we developed a treatment strategy for anti-GBM glomerulonephritis. AAV2-GEC vectors carrying secretory *IdeS* (see Methods) and *GFP* under the control of the CMV promoter were used as treatment and control vectors, respectively.

**Figure 5:**
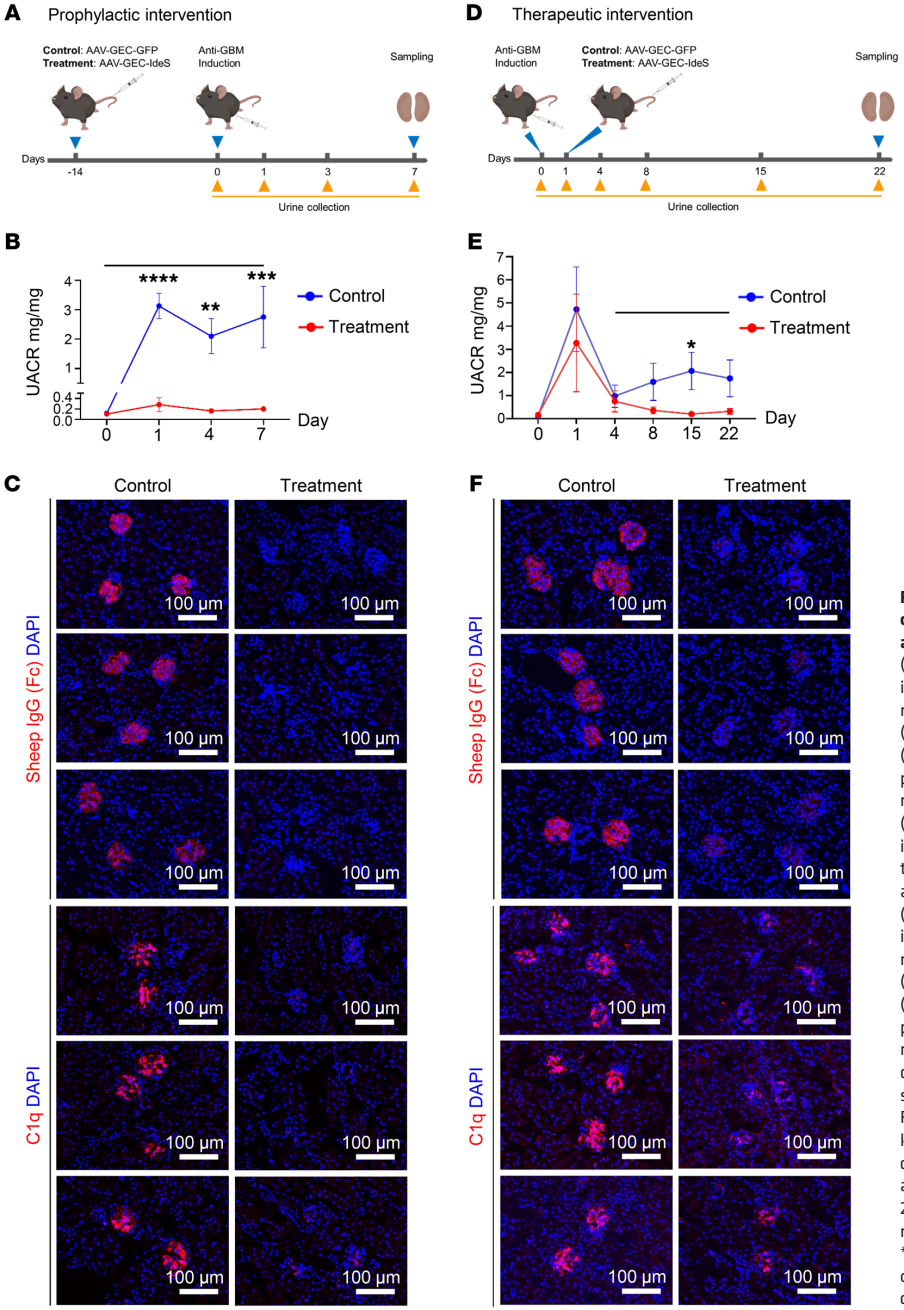
AAV2-GEC delivery of IdeS successfully treated anti-GBM glomerulonephritis. (**A**) Schematic of the prophylactic intervention protocol. C57BL/6J mice were divided into control (AAV2-GEC-GFP) and treatment (AAV2-GEC-IdeS) groups. *n* = 10 per each group. (**B**) UACR was measured at 0, 1, 3, and 7 days. (**C**) Representative images showing remaining sheep IgG Fc and the deposition of C1q in kidneys at 7 days. Scale bars: 100 μm. (**D**) Schematic of the therapeutic intervention protocol. C57BL/6J mice were divided into control (AAV2-GEC-GFP) and treatment (AAV2-GEC-IdeS) groups. *n* = 6 per each group. (**E**) UACR was measured at 0, 1, 4, 8, 15, and 22 days. (**F**) Representative images showing remaining sheep IgG Fc and the deposition of C1q in kidneys at 22 days. Nuclei were counterstained with DAPI. Values are mean ± SEM. Significance: 2-way ANOVA with repeated measures, *P < 0.05, **P < 0.01, ***P < 0.001, ****P < 0.0001; only statistically significant comparisons are shown.

For the prophylactic interventions, the treatment and control vectors were intravenously injected in adult C57BL/6J male mice with a dose of 1 × 10^13^ vg/kg. Two weeks after AAV injection, all mice received 150 μl anti-GBM serum produced in sheep to induce glomerulonephritis (Figure 5A).

To monitor the GFB function, the urinary albumin-to-creatinine ratio (UACR) was measured during the progression of anti-GBM glomerulonephritis (Figure 5B). On day 1 after anti-GBM serum injection, albuminuria was detected in control mice, which was persistent from day 3 until day 7. In contrast, the onset of albuminuria was prevented in treated mice. Only a very mild increase in UACR was measured at day 1, which declined to baseline at day 3 and was maintained at the low level until day 7. These results suggest that delivery of the AAV2-GEC-IdeS efficiently prevented albuminuria in the progression of anti-GBM glomerulonephritis.

IdeS specifically cleaves IgG in the hinge region, yielding the Fab and Fc fragments (25). Kidney IF sections showed that the Fc fragments of sheep IgG were barely detectable in treated mice but predominantly deposited on the GBM of control mice (Figure 5C). The accumulation of sheep and mouse IgG on the GBM was also strongly reduced in treated mice (Supplemental Figure 7A), which then substantially reduced the deposition of complement C1q and C3 (Figure 5C and Supplemental Figure 7A).

Next, we evaluated the therapeutic potential of AAV2-GEC-IdeS by injecting the treatment and control vectors 1 day after the induction of anti-GBM glomerulonephritis (Figure 5D). While both control and treated mice experienced an initial peak in albuminuria on day 1, the subsequent course differed. Control mice displayed decreased UACR but albuminuria persisted until day 22, while treated mice exhibited a marked decrease in UACR, reverting to baseline levels from day 8. This differential response suggests a successful therapeutic effect of AAV2-GEC-IdeS in mitigating albuminuria (Figure 5E).

Kidney IF sections showed that the Fc fragments of sheep IgG, as well as the complement C1q and C3 were barely detectable in treated mice but predominantly deposited on the GBM of control mice (Figure 5F and Supplemental Figure 7B). The deposition of sheep and mouse IgG on the GBM was weaker in treated mice than in control mice (Supplemental Figure 7B). These results suggest that delivery of the AAV2-GEC-IdeS after anti-GBM glomerulonephritis also efficiently prevented albuminuria and the disease progression.

Additionally, serum liver function indexes (ALTL, ASTL, GGT-2, CHOL2, TRIGL, and BILD2) were normal in both treated and control mice (Supplemental Figure 8), indicating no liver toxicity caused by AAV2-GEC-IdeS injection.

The long-term expression of secretory IdeS was analyzed by monitoring its serum level for 240 days (Supplemental Figure 9). IdeS was fused with nanoluciferase (Nluc) and delivered to GEC by intravenous injection of AAV2-GEC-IdeS-Nluc with a dose of 5 × 10^12^ vg/kg. The concentration of circulating IdeS was maintained from day 3 until day 240, indicating a stable expression of IdeS by transduced GEC.

Taken together, these results suggest that AAV2-GEC-IdeS transduction efficiently produced IdeS in GEC, which provided sustained clearance of kidney-bound IgG and successfully prevented the progression of anti-GBM glomerulonephritis.

## Discussion

Discovering new AAV vectors with cell-targeting properties plays an important role in developing new therapeutic approaches for kidney disease. AAV capsid engineering by directed evolution allows the generation of diverse capsid libraries, and the iterative selection of such libraries enables the identification of AAV vectors with desired tropisms. This strategy has been successfully applied to several organs and tissues (15, 16, 26–34), but less progress has been made in the kidney.

In this study, we tailored the selection process for the kidney to identify GFB-targeting AAV vectors. Anatomically, the kidney can be divided into the glomerular and tubular compartments, which exert filtration and reabsorption functions, respectively. The tubular compartment contains mainly epithelial cells that form different tubular segments, whereas the glomerular compartment contains cell types other than epithelial cells, including EC, mesangial cells, and podocytes, the latter being nontypical epithelial cells with contractile characteristics (35). As the cells in these 2 compartments are distinct in type, it would have been unlikely to identify glomerulus-targeting AAVs if the whole kidney had been used as the target throughout the whole selection process. Therefore, we used the whole kidney as the target in the first 2 rounds of selection to ensure that the next round of AAV libraries included all relevant AAV clones with kidney selectivity. We then increased the target resolution by using isolated glomeruli as targets in the following 2 selection rounds to further enrich the AAV variants with desired specificity. This strategy of increasing the selection pressure over the selection rounds enabled a steady enrichment of truly specific AAVs. Meanwhile, we monitored the selection dynamics and discontinued the selection process once the library variability was obviously decreased. However, NGS analyses showed that the diversity of recovered AAV variants was still large at this point. To quantitatively evaluate the AAV variants with high transduction efficiency and specificity, we used a scoring analysis that calculates not only the relative abundance in the target tissue (E score) but also the distribution in the off-target organs (GS score). The combination of these 2 factors (C score) was an important criterium for the selection in this study. As a result, QVLVYRE was selected as the most promising AAV variant, which indeed exhibited strong transduction efficiency and specificity for the glomerulus as the target tissue of this library selection.

After systemic administration, AAVs circulate into the kidney via the renal artery, reach the nephron and enter the glomerulus via the afferent arteriole, then reach the tubular segments via the peritubular capillaries or the descending vasa recta branching from the efferent arteriole of the glomerulus (36). The renal vasculature is extremely complex with high heterogeneity and plasticity, and it has the most diverse endothelial cell types of any organ (37). Single-cell transcriptomics identifies 7 major vascular clusters in the adult mouse kidney, including the GEC from large arteries and the afferent arteriole, the GEC and the postglomerular EC from the efferent arteriole, descending vasa recta, peritubular capillaries, ascending vasa recta, venous blood vessels, and progenitor EC (38). When considering the cortical, medullar, and glomerular distribution of renal EC, which contribute to different physiological functions, they are subclustered into 24 subpopulations with distinct marker genes (36). GEC are distinguishable from other renal EC types by canonical marker genes such as EH domain–containing 3 (EHD3), which regulates the endocytic recycling of vascular endothelial growth factor receptor 2 (VEGFR2) (39) and maintains the GEC fenestration (20). Unlike the typical fenestrated EC from the peritubular capillaries and ascending vasa recta of the kidney or other organs, GEC lack the plasmalemma vesicle associated protein–containing (PLVAP-containing) diaphragms (40). Instead, the luminal side of the GEC fenestrae is covered by exceptionally rich and diverse glycoproteins, proteoglycans, and glycosaminoglycans of the glycocalyx, which is functionally essential for the maintenance of charge selectivity and the regulation of GFB permeability (2). Hence, GEC are regarded as unique fenestrated endothelium by expressing differential marker genes and possessing specialized glycocalyx, both of which can in turn play a critical role in AAV binding and internalization (41). It is not yet clear which glycans and plasma membrane proteins are critical to the GEC-targeting of AAV2-GEC. Comprehensive and comparative omics studies to better characterize GEC at multiple levels will be helpful to address this question in the future.

AAV2-GEC maintained robust tropism in C57BL/6J, Balb/c and BTBR mouse strains and in SD rats. It also maintained robust tropism under different pathological conditions. Further, it exhibited enhanced transduction in human primary GEC compared with AAV2-WT, but not in podocytes or mesangial cells. Interestingly, AAV2-GEC exhibited comparable transduction of the 3 glomerular cell types in vitro. It is important to note that in vitro experiments do not necessarily reflect the in vivo situation as the cell surface composition differs between in vivo and in vitro environments, which may affect AAV targeting. In addition, systemic delivery of AAV results in uneven distribution within organs, particularly the kidney, where the GFB restricts AAV access to podocytes. As a result, AAV, which generally shows poor transduction in EC, fails to transduce podocytes after systemic administration. These reasons might explain the in vitro observations.

It is well known that EC exhibit significant differences in gene expression between species even in the identical vasculature (37). These potential interspecies differences may result in AAV variants selected in a particular mouse strain not expanding to other strains or species or translating to humans (37). Importantly, EC gene expression differs under physiological and pathological conditions. In the kidney, GEC are sensitive to proinflammatory factors, prothrombotic mediators, and the disruption of glycocalyx (18). DKD is a typical chronic kidney disease that causes damage to GEC. Hyperglycemia, oxidative stress, and binding of advanced glycation end product (AGE) promote glycocalyx disruption and result in the loss of GEC fenestrae (18). In addition, the intercellular crosstalk between podocytes and GEC contributes to the pathogenesis of various glomerular diseases (42). In particular, abnormal secretion of VEGF by stressed podocytes leads to GEC dedifferentiation and dysfunction (42, 43). Thus, the DKD and podocyte injury models, which cause marked changes in GEC phenotypes and also their tropisms, are suitable to evaluate the targeting specificity of AAV2-GEC in pathological settings. Our results indicate that AAV2-GEC has conserved GEC tropism under both physiological and pathological conditions at least in rodents. It is important to test the targeting specificity of AAV2-GEC in larger animals and nonhuman primates to evaluate its translational potential in the future.

We monitored the AAV2-GEC–mediated GFP expression over 360 days and showed that the GFP signal decreased significantly after 240 days. Since AAV vectors largely persist as nonintegrated episomal DNA potentially getting diluted or lost upon cell division (44), our results indicate that GEC have a long turnover rate in healthy kidneys, which has also been suggested by other studies (45, 46). However, to our knowledge, no studies have specifically determined the turnover rate of GEC in healthy conditions. In pathological conditions such as hypertension and hyperglycemia, proliferation and remodeling of GEC have been observed, although the turnover rate remains undefined (47). We evaluated the transduction efficiency of AAV2-GEC in healthy and pathological conditions and found that GFP expression in GEC was not statistically significantly different between healthy and pathological conditions. Interestingly, AAV2-GEC transduction did not significantly alter the GEC transcriptomics in C57BL/6J, but did in BTBR*^ob/ob^* and *Nphs1*^ΔiPod^ mice. Bioinformatics analysis suggests that AAV2-GEC transduction reduced ribosome biogenesis and RNA binding in GEC under pathological conditions. Ribosome biogenesis is fundamental to normal cell growth, development, and differentiation, and its dysregulation can lead to a variety of diseases. Viruses are generally thought to be able to interact with the nucleolus and its components involved in ribosome biogenesis in infected cells, thereby promoting viral replication (48). It is important to note that AAV is a replication-defective virus. Whether the phenomenon observed under pathological conditions was due to AAV transduction or AAV-delivered transgene expression needs to be further investigated.

GEC are a primary target for therapeutic interventions in kidney genetic diseases, as well as in the context of antiinflammation, inhibition of coagulation, and protection of glycocalyx in glomerular diseases (5, 18, 43). Many gene defects have been identified in GEC. Most of these genes participate in the complement pathway underling the pathogenesis of atypical hemolytic uremic syndrome (aHUS) and C3-dominant glomerulopathy (4, 49). Mutations in factor H (*CFH*), factor I (*CFI*), C3, factor B (*F8*), membrane cofactor protein (*MCP*), and thrombomodulin (*THBD*) contribute to 50% of all aHUS cases (50). Two noncomplement genes, diacylglycerol kinase epsilon (*DGKE*) and inverted formin 2 gene (INF2), can cause childhood onset of aHUS, which are also related to steroid-resistant nephrotic syndrome (51, 52). GEC play an essential role in maintaining the GFB, actively interact with podocytes and mesangial cells,and directly contact circulating factors from the bloodstream (18, 40). Therefore, GEC are a therapeutic target to improve their own cellular functions, such as the modification of gene defects and the preservation of glycocalyx, which is a key for the treatment of DKD and focal segmental glomerulosclerosis (40). Importantly, GEC can also be used as a biofactory to produce and distribute therapeutic molecules such as enzymes to intervene in the functionality of neighboring cells or to prevent pathological processes in glomeruli. This concept could be especially useful for the treatment of kidney autoimmune diseases such as lupus nephritis, IgA nephropathy, and anti-GBM disease (43). Since inflammation and coagulation are common complications in the kidney leading to a decline in glomerular filtration rate and ultimately kidney failure, efficient removal of pathogenic antibodies depositing on the GFB is critical for the preservation of kidney function (43).

To prove the feasibility of this concept, we used AAV2-GEC to deliver IdeS in the GEC for the treatment of glomerulonephritis. IdeS is a streptococcal IgG-degrading enzyme that showed remarkable ability in cleaving circulating antibodies in experimental anti-GBM glomerulonephritis (53) and has been tested in a phase II trial in severe anti-GBM disease (14). Anti-GBM glomerulonephritis is a subtype of autoimmune glomerulonephritis caused by the presence of antibodies against the type IV collagen α 3 chain in the GBM (43). Removal of kidney-bound and circulating antibodies at a rapid pace is essential for the treatment of anti-GBM glomerulonephritis to prevent the progression to end-stage kidney failure (54). Our experiments showed that AAV-delivered IdeS, which was stably produced by the GEC, efficiently cleaved the pathological IgG and successfully prevented the progression of anti-GBM glomerulonephritis.

Compared with the 1-dose infusion of IdeS in patients, which cleaves and removes IgG within 6 hours (14), AAV2-GEC–mediated IdeS production in mice took approximately 3 days, and the serum concentration of IdeS peaked at 7 days after intravenous injection. It is important to note that, unlike in humans, experimental anti-GBM glomerulonephritis induces transient phenotypes and presents a recovery course in mice (53). Our results showed that AAV2-GEC-IdeS injection before or after the onset of anti-GBM glomerulonephritis prevented or ameliorated albuminuria. Kidney IF results suggested that when AAV2-GEC-IdeS was injected before the onset of glomerulonephritis, GEC-secreted IdeS efficiently cleaved the circulating anti-GBM IgG, thereby preventing the deposition of IgG on the GBM. In contrast, when AAV2-GEC-IdeS was injected after the onset of glomerulonephritis, anti-GBM IgG was already bound to the GBM. GEC-secreted IdeS also efficiently cleaved the GBM-bound IgG, removing the Fc fragments while leaving the GBM-bound Fab fragments. Nevertheless, the sufficient removal of Fc prevented the activation of the complement system. Therefore, our study shows that the concept of using AAV2-GEC and GEC-derived transgenic expression is feasible for the treatment of antibody-mediated kidney diseases.

Notably, 1-dose infusion of IdeS cannot prevent the occurrence of rebound antibodies, and the majority of patients will need additional sessions of plasma exchange or immunoadsorption after 1 week (14, 54). A second dose of IdeS is currently not suggested due to the concerns for anti-IdeS antibodies and immune complex-mediated hypersensitivity (13, 14, 54). Interestingly, in AAV2-GEC-IdeS–injected mice, the serum IdeS was maintained at a comparable level measured at 7 days for over 240 days, suggesting that the GEC-secreted IdeS was not neutralized by antibodies and the AAV-transduced GEC were not eliminated by the immune system. Moreover, the prolonged and sustained expression of IdeS mediated by GEC may provide a solution to remove rebound antibodies. For the translational use in the future, engineering works will be useful to enable the precise regulation of the cargo gene expression in pathological settings to overcome side effects such as sustained and irreversible immunosuppression. Nonetheless, we believe that this proof-of-concept experiment suggests the therapeutic potential of AAV2-GEC, which targets GEC not only for kidney genetic diseases but also for multiple other kidney diseases.

In conclusion, this study establishes an AAV in vivo screening approach for renal glomeruli. It identifies a novel GEC-targeting AAV vector with robust tropism maintained across species in both physiological and pathological settings. The identification of AAV-GEC demonstrates the feasibility of future GFB-targeting strategies for novel kidney therapies.

## Methods

### Sex as a biological variable.

Our study exclusively examined male mice. It is unknown whether the findings are relevant for female mice.

### Animals.

BTBR*^ob/+^* (BTBR.Cg-Lep^ob/wt^ ^WiscJ^) was purchased from the Jackson Laboratory (Jax No. 004824) to generate BTBR*^ob/ob^* mice. *Neph1*-floxed mice were crossed with Tg(Nphs1-rtTA*3G)^8Jhm^ and Tg(tetO-cre)1^Jaw^ to generate *Nphs1*^ΔiPod^ mice (21). Doxycyclin (2 mg/mL in 5% sugar solution, Fagron) was administered for 7 days in the drinking water at an age of 5 weeks. C57BL/6J and Balb/c mice were purchased from Charles River. SD rats were purchased from Envigo. Intravenous administration mentioned in all experiments was done via tail vein. Animals were housed in a specific pathogen-free facility with free access to food and water and were kept on a 12 hour light/dark cycle. Breeding and genotyping were performed according to standard procedures.

### Preparation of the AAV2 display peptide library.

A random AAV2 display peptide library plasmid with a diversity of 1.5 × 10^8^ unique clones was produced as previously described (17). In short, the degenerate oligonucleotides of 7 random amino acids (encoded by Trimer technology leading to no codon bias and no production of stop codons) was synthesized commercially as follows: 5′-CAGTCGGCCAGAGAGGC-(Trimer)_7_-GCCCAGGCGGCTGACGAG-3′ (Ella Biotech). The second strand was synthesized using the sequenase (Thermo Fisher Scientific, 70775Y200UN) and the primer 5’-CTCGTCAGCCGCCTGG-3’. The double-stranded oligonucleotide insert was cleaved with BglI and ligated into the SfiI-digested pMT202-6 library plasmid at nucleotide position 3,967 of the AAV genome (55). The diversity of the plasmid library was determined by the number of large-scale transformed clones on LB agar plates. Library plasmids were harvested from transformed bacteria and purified using QIAGEN Plasmid Maxi Kit (QIAGEN, 12163). The AAV2 display peptide primary library was produced by transfection of 1 × 10^9^ HEK293T/17 cells (ATCC, CRL-11268) in 100 150-mm cell culture dishes. A 1-step procedure that allows the production of highly diverse AAV libraries to keep the maximal capsid-genome correlation of AAV particles was adopted. For each plate, 100 ng library plasmid (equal to 500 plasmids per cell) and 11.9 μg pXX6 plasmid were cotransfected with PolyFect Transfection Reagent (Qiagen, 301107) (12, 56, 57). Three days after transfection, the AAV particles were harvested and subsequently purified by iodixanol density-gradient ultracentrifugation. The virus titer was determined by real-time PCR using cap-specific primers (17) and titers are expressed as viral genomes per mL (vg/mL).

### AAV in vivo screening in the kidney.

Initial AAV2 peptide library was injected with 7.5 × 10^10^ vg per mouse (C57BL/6J, 20 g), subsequent libraries were injected with 1 × 10^11^ vg per mouse (C57BL/6J, 20 g). At 3 or 6 days postinjection, mice were sacrificed and kidneys or glomeruli and off-target organs were harvested, respectively. Four screening rounds of the library were performed in vivo. In the first 2 rounds of screening, the AAV library fragments were rescued from the genomic DNA of the whole kidney. To increase the selection pressure, AAV library fragments were rescued from genomic DNA extracted from glomeruli in the third and fourth rounds of screening. Sublibrary plasmids were generated for the next round of selection as described previously (12). In brief, the random heptamer oligonucleotides of the AAV library particles from the enriched kidney were amplified by nested PCR with the primers 5′-ATGGCAAGCCACAAGGACGATG-3′ and 5′-CGTGGAGTACTGTGTGATGAAG-3′ for the first PCR, and 5′-GGTTCTCATCTTTGGGAAGCAAG-3′ as well as 5′-TGATGAGAATCTGTGGAGGAG-3′ for the second PCR (optional). The PCR-amplified heptamer oligonucleotides were subcloned into the library plasmid pMT202-6 (55). The sublibraries were produced like the primary library described above but only transfect 1 × 10^8^ HEK293T/17 cells in 10 150-mm cell culture dishes.

### Renal glomeruli isolation.

The glomeruli isolation from the mouse kidney has been described previously (19). In brief, the kidney was perfused with 2 mL warm Dynabeads (Invitrogen) via the renal arteries ex vivo. After perfusion, kidney papilla and capsule were removed and minced into small pieces on ice. Tissues were then digested and homogenized in 5.5 mL collagenase V solution for 10 minutes at 37 °C and homogenized every 5 minutes using a gentleMACS dissociator (Miltenyi Biotec). Homogenized tissues were passed through the 300 μm cell strainer followed by a 100 μm cell strainer and washed with ice-cold 1 × HBSS. Tissue suspension was centrifuged and the pellet was washed over 3 times on the magnet. Glomeruli were pooled for genomic DNA extraction.

### Plasmid construction.

The IdeS gene (accession number JN035367) with the secreted signal peptide of albumin was synthesized at Thermo Fisher Scientific. The synthesized fragment was cloned into the vector pscAAV-CMV-GFP (Addgene, plasmid 32396) by replacing the GFP with AgeI and BsrGI to generate pscAAV-CMV-IdeS vectors. All AAV-packaged transgene payloads in the study were in self-complementary form.

### Vector production and quantification.

Recombinant AAV vectors were produced by cotransfection of HEK293T/17 cells with 16 μg of the pXX6 helper plasmid (55), 8 μg of the Rep/Cap plasmid, and 8 μg of the AAV2 ITR-flanked transgene plasmid using polyethylenimine (Polysciences, linear). Recombinant virus was harvested from the cells and media and purified by iodixanol gradient ultracentrifugation as previously described (12). AAV titers were quantified by qPCR with the forward primer 5′-GGGACTTTCCTACTTGGCA-3′ and the reverse primer 5′-GGCGGAGTTGTTACGACAT-3′ directed to the CMV promoter sequence.

### NGS sample preparation and bioinformatics.

For the AAV library NGS sample preparation, 3 steps of PCR amplification were performed. In the first step, enriched viral DNA was amplified from target and off-target organs by the nested PCR described above. In the second PCR, linker sequences with the forward primer 5′-ACACTCTTTCCCTACACGACGCTCTTCCGATCTGCTCCAGAGAGGCCAGAGAG-3′ and the reverse primer 5′-TGACTGGAGTTCAGACGTGTGCTCTTCCGATCTATGAGCATCTGCGGTGGCCGCCTG-3′ were attached to the viral DNA fragment (12). And an individual Illumina barcode was introduced to each sample with the forward primer 5′-AATGATACGGCGACCACCGAGATCTACACTCTTTCCCTACACGAC-3′ and the reverse primer 5′-CAAGCAGAAGACGGCATACGAGAT (7nt_index) GTGACTGGAGTTCAGACGTGTG-3′ in the third PCR. The samples labeled by individual Illumina barcodes were pooled in a 150 μl mixture with 2 nmol/L per sample. Subsequently, the size and quality of DNA were assessed by Agilent 2100 Bioanalyzer system according to the manufacturer’s recommendation. NGS and demultiplexing were performed on an Illumina MiSeq sequencer (600-cycle, single-indexed, paired-end run) with MiSeq Reagent Kit v3 (MS-102-3003, Illumina). Approximately 100,000 reads per sample were yielded. All primers mentioned above were purchased from Thermo Fisher Scientific.

Known, invariable flanking sequences of length 10 bp (CCAGAGAGGC and GCCCAGGCGG) were used to extract only insert sequences of target length (21 bp) from the sequence reads. Reads not matching these flanking sequences exactly and reads with diverging insert sizes were removed from the analysis. Nucleotide insert sequences for which there were at least 100-fold more frequent insert sequences within an edit distance of 1 were considered possible artifacts and removed. Moreover, insert sequences with codons not matching the expected coding pattern (Trimer) were removed and the remaining sequences were translated into peptides.

### Score (GS, E, and C) and off-target organs.

The AAV library–enriched NGS data was evaluated by rating scores as described previously (16). The enrichment score ‘E’ was used to evaluate transduction efficacy in the target of each candidate reflecting changes in relative abundance from before-last to last selection. The general tissue specificity score ‘GS’ was used to assess the tropism of each candidate among target organs and other off-target organs (liver, heart, lung, muscle, brain, pancreas, and spleen) by multiplying the individual specificity scores (S_liver_ × S_heart_ × S_lung_ ×...). To determine the most promising candidate regarding specificity and efficacy, a combined score ‘C’, was determined by multiplying E and GS. To calculate E and S scores, the following formula was used:

 (Equation 1)
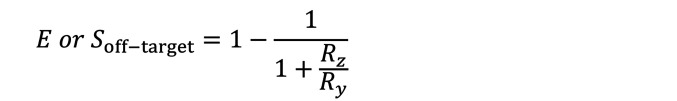


where E score is the relative abundances third round (Ry) and fourth round (Rz) in target tissues and S_off-target_ score is the relative abundances of fourth round off-target (Ry) and the same round target (Rz).

### Vector genome distribution analysis.

Fourteen days after AAV injection, the biodistribution of the AAV vectors containing the GFP gene was studied by quantifying GFP transgene copy numbers in the isolated glomerulus and other relevant organs. After DNA extraction, 100 ng gDNA from each sample was analyzed by qPCR with the GFP-specific forward primer: 5′-CTACGGCGTGCAGTGCTTCAG-3′ and the reverse primer: 5′-CTTCAGCTCGATGCGGTTCAC-3′. The number of vector genomes was quantified and normalized to vector copy numbers per diploid genome (vg/dg). The corresponding plasmid (pscAAV-CMV-GFP) was serially diluted and used as a standard curve.

### Treatment of anti-GBM glomerulonephritis by AAV-GEC–delivered IdeS.

8 week old C57BL/6J male mice with comparable body weights were randomly assigned to 2 groups. For the prophylactic intervention, 1 group was injected with AAV2-GEC-IdeS and the other group was injected with AAV2-GEC-GFP intravenously at a dose of 1 × 10^13^ vg/kg. At 14 days after AAV injection, both groups were injected with 150 μl of anti-GBM serum (PTX-001 sheep anti-rat GBM serum, Probetex Inc.) by intraperitoneal injection. Urine was collected before and 1, 3, and 7 days after anti-GBM serum injection. Animals were sacrificed 7 days after anti-GBM serum injection. For the therapeutic intervention, both groups were injected with 150 μl of anti-GBM serum by intraperitoneal injection. The next day, 1 group was injected with AAV2-GEC-IdeS and the other group was injected with AAV2-GEC-GFP intravenously at a dose of 1 × 10^13^ vg/kg. Urine was collected before and 1, 4, 8, 15, and 22 days after the anti-GBM serum injection. Animals were sacrificed 22 days after the anti-GBM serum injection.

### IF.

Each mouse was perfused with 5 mL DPBS followed by 5 mL 4% PFA and the tissues were subsequently fixed in 4% PFA at 4 °C overnight. For the IF, kidney samples were incubated in 15% sucrose in PBS at 4°C for 6 hours followed by 30% sucrose in PBS overnight and embedded in OCT. Cryosections were permeabilized with 0.1% Triton X-100. Nonspecific binding sites were blocked in 5% BSA supplemented with 0.3M Glycine in PBST. Cryosections were incubated overnight at 4°C with the following primary antibodies: chicken anti-GFP, 1:500 (Thermo Fisher Scientific, A10262); rat anti-CD31, 1:200 (BD Pharmingen, 550274); rat anti-CD16/CD32, 1:200 (Cell Signaling Technology, 88280S); rabbit anti-WT1, 1:200 (Abcam, ab89901); rabbit anti-PDGFR, 1:200 (Abcam, ab32570); mouse anti-goat/sheep IgG-Biotin (Fc-specific), 1:1,000 (Sigma-Aldrich, B3148); Alexa Fluor 555 donkey anti-sheep IgG, 1:1,000 (Invitrogen, A21436); Alexa Fluor 555 donkey anti-mouse IgG, 1:1,000 (Invitrogen, A31570); Mouse anti-RECA-1, 1:200 (Novus Biologicals, NB600-1388); rabbit anti-C1q, 1:200 (Abcam, ab182451); rat anti-C3, 1:200 (Novus Biologicals, NB200-540SS). Alexa Fluor 555 conjugated Streptavidin and Alexa Fluor 488-, 594-, or 647-labeled secondary antibodies (Invitrogen) were incubated at room temperature for 1 hour. DAPI was used for staining nuclei. Images were taken using Zeiss Apotome and Leica SP5 or SP8 microscope.

### Statistics.

Data represent mean + SD or mean ± SEM and were analyzed using 1-way ANOVA for multiple comparisons with Dunnett’s test (Figure 1D ), or 2-way ANOVA with repeated measures (Figure 5, B and E). *P* values of less than 0.05 were considered significant. Statistical analyses were performed using GraphPad Prism 8.

### Study approval.

All animal procedures were performed in compliance with the National Institutes of Health Guide for the Care and Use of Laboratory Animals as well as the German law for the welfare of animals. Animal experiments were approved by the veterinary administration of the City of Hamburg under the license N054-2018, N091-2020, and N089-2021.

### Data availability.

Sequence data reported in this publication have been submitted to the European Nucleotide Archive (ENA). They are publicly available under accession PRJEB76952. All supporting values for this manuscript are provided in the Supporting Data Values file.

## Author contributions

TBH initiated the project. S. Liu, S. Lu, JK, and TBH designed the study. GW, S. Liu, S. Lu, and JK established the methodology. S. Liu and S. Lu administrated the project. GW, S. Liu, JH, MA, MS, NA, ZL, AVF, JD, JK, and S. Lu performed the experiments and analyzed the data. S. Liu wrote the original manuscript. GW, S. Liu, JH, MA, FEH, MS, NA, ZL, NW, NMT, AVF, JD, JK, S. Lu, and TBH reviewed and edited the manuscript. All authors approved the final version of the manuscript.

## Supplementary Material

Supplemental data

Supplemental table 1

Supporting data values

## Figures and Tables

**Table 2:**
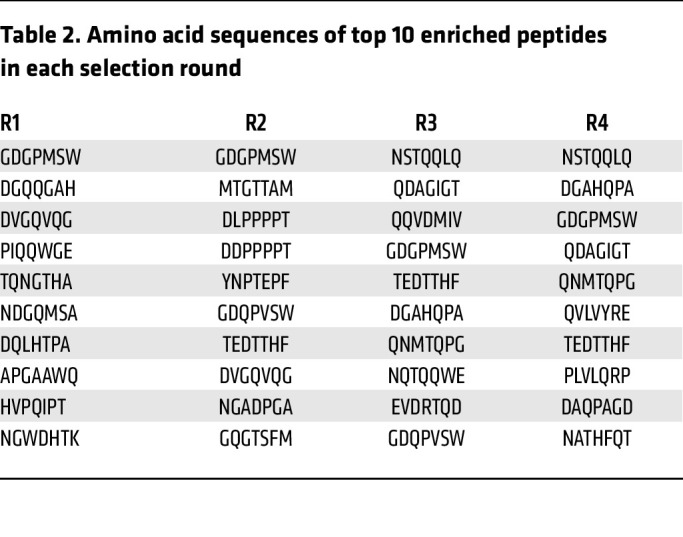
Amino acid sequences of top 10 enriched peptides in each selection round

**Table 1:**
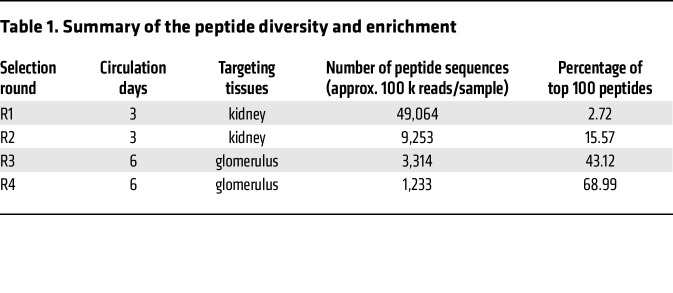
Summary of the peptide diversity and enrichment

## Version 1 09/03/2024

Electronic publication
