# Supplementary material for: Adeno-associated virus–based gene therapy treats inflammatory kidney disease in mice
Source: J Clin Invest. 2024 Aug 15;134(17):e174722. doi: 10.1172/JCI174722 (PMC11364381; doi:10.1172/JCI174722)
Supplement: Supplemental data [file jci-134-174722-s131.pdf]

Supplemental material

Adeno-associated virus-based gene therapy treats inflammatory kidney disease in mice

Guochao Wu[1, 2, §], *Shuya Liu*[1, 2, §, *], *Julia Hagenstein*[1, 2], *Malik Alawi*[3], *Felicitas E. Hengel*[1, 2], *Melanie Schaper*[1, 2], *Nuray Akyüz*[4], *Zhouning Liao*[1, 2], *Nicola Wanner*[1, 2], *Nicola M. Tomas*[1, 2], *Antonio Virgilio Failla*[5], *Judith Dierlamm*[4], *Jakob Körbelin*[4, #], *Shun Lu*[1, 2, #], *Tobias B. Huber*[1, 2, #, *]

[1]III. Department of Medicine, University Medical Center Hamburg-Eppendorf, Hamburg, Germany.
[2]Hamburg Center for Kidney Health (HCKH), University Medical Center Hamburg-Eppendorf, Hamburg, Germany
[3] Bioinformatics Core, University Medical Center Hamburg-Eppendorf, Hamburg, Germany.
[4] Department of Oncology, Hematology and Bone Marrow Transplantation with Section Pneumology, University Medical Center Hamburg-Eppendorf, Hamburg, Germany.
[5]Microscopy Imaging Facility, University Medical Center Hamburg-Eppendorf, Hamburg, Germany.

[§, #]These authors contributed equally to this work.

*Corresponding author.

Correspondence information:

Shuya Liu, PhD
Email: s.liu@uke.de
Telefone: +49 (0)40 7410-51911

Tobias B. Huber, MD
E-mail: t.huber@uke.de
Telefone: +49 (0)40 7410-53908

III. Department of Medicine
University Medical Center Hamburg Eppendorf
Martinistr. 52
20246 Hamburg

Supplemental Figures

Supplemental figure 1

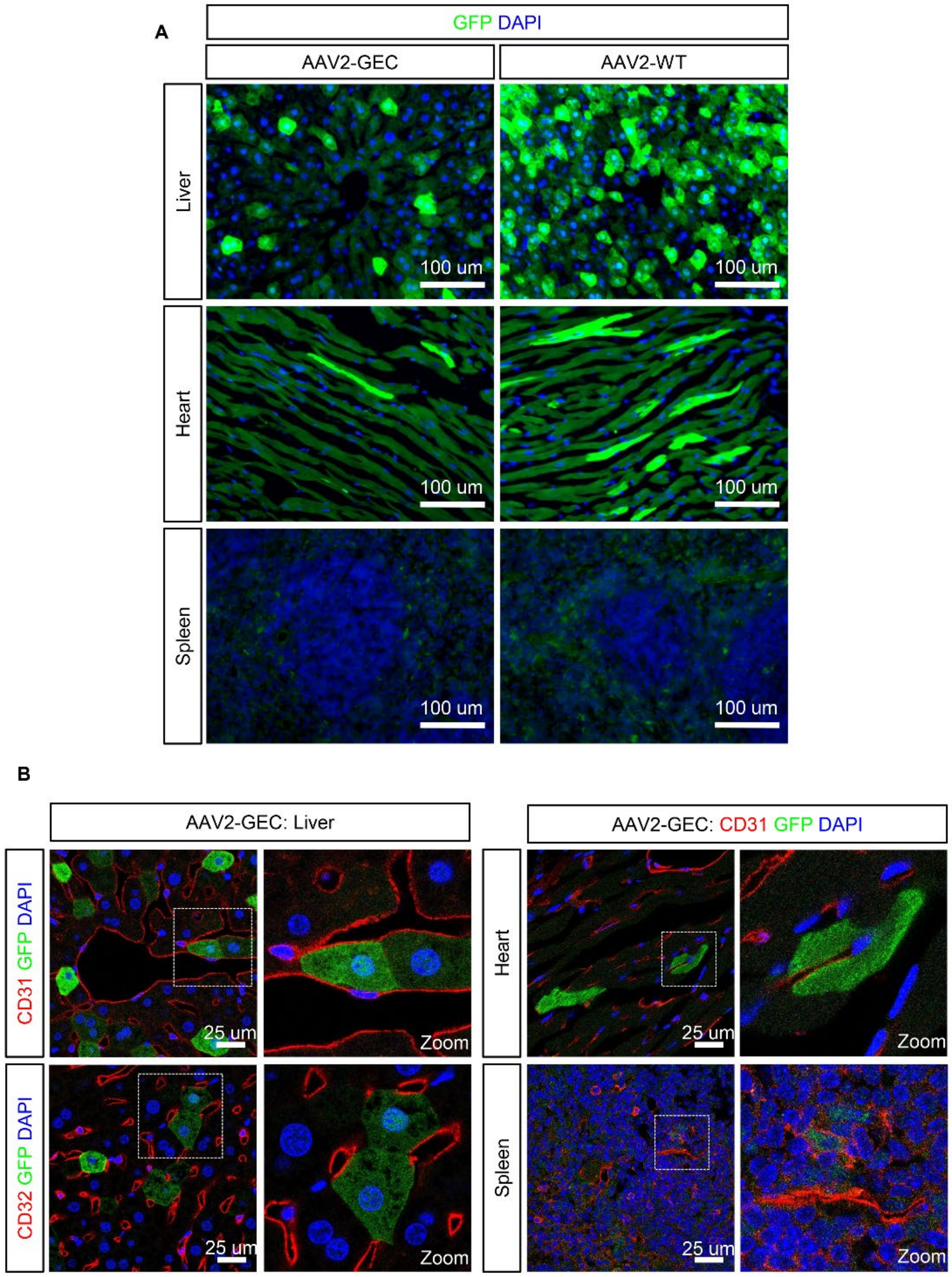

Supplemental Figure 1. AAV2-GEC mediated GFP expression in off-target organs

(**A**) Representative images of GFP expression in the off-target organs (liver, heart and spleen) delivered by AAV2-GEC compared to AAV2-WT. (**B**) AAV2-GEC-mediated GFP expression was not detected in endothelial cells marked by anti-CD31 antibody in the liver, heart and spleen, and not in the liver sinusoidal endothelial cells marked by anti-CD32 antibody. Nuclei were counterstained with DAPI.

Supplemental figure 2

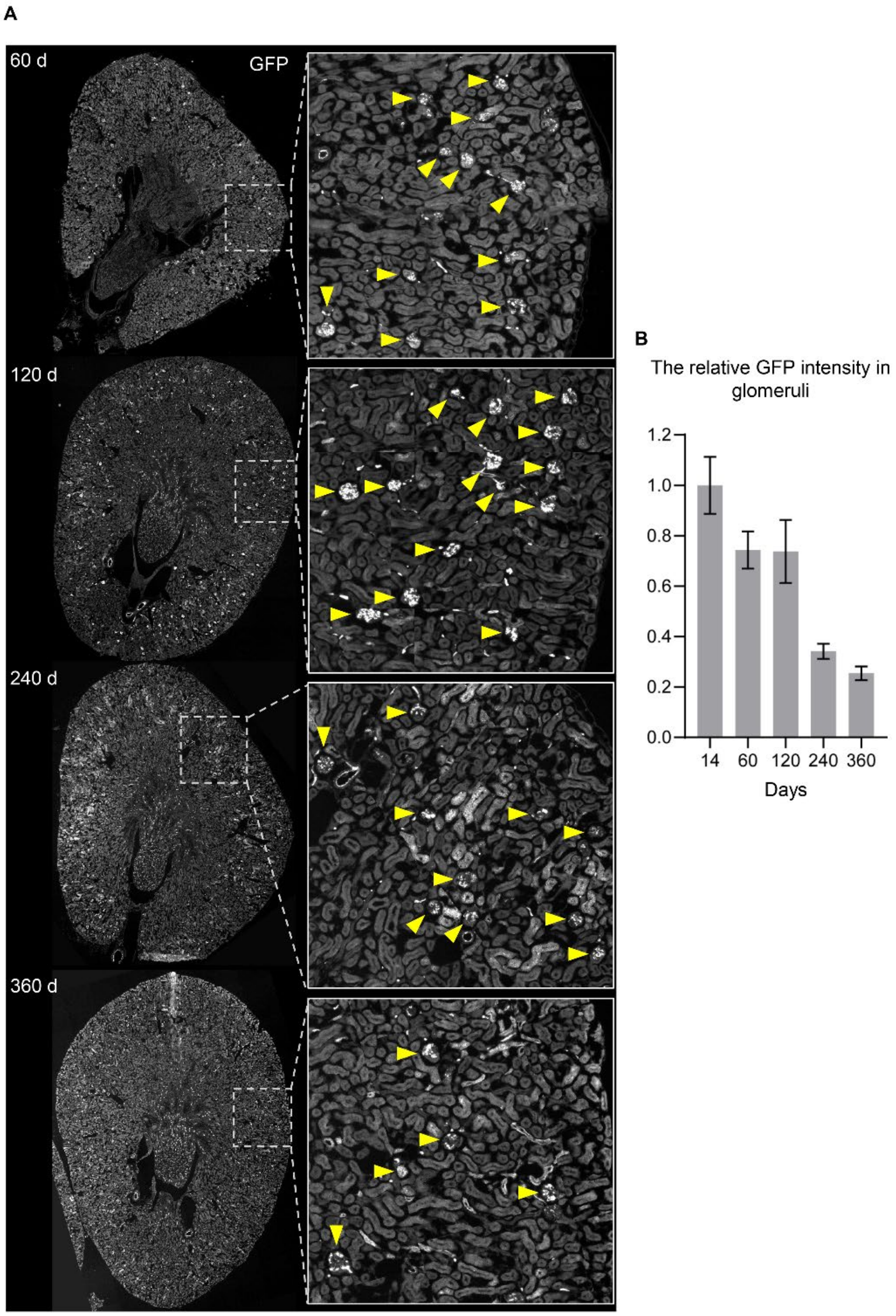

Supplemental Figure 2. AAV2-GEC mediated long-term GFP expression in the kidney

(**A**) Representative overview images of AAV2-GEC mediated GFP expression in kidneys from C57BL/6J at different time points after intravenous injection. Arrows indicate GFP-positive glomeruli. (**B**) Quantification of GFP signal intensity in glomeruli at different time points. N=2 per time point and 4 kidneys were analyzed in total. Values are mean ± SEM.

Supplemental figure 3

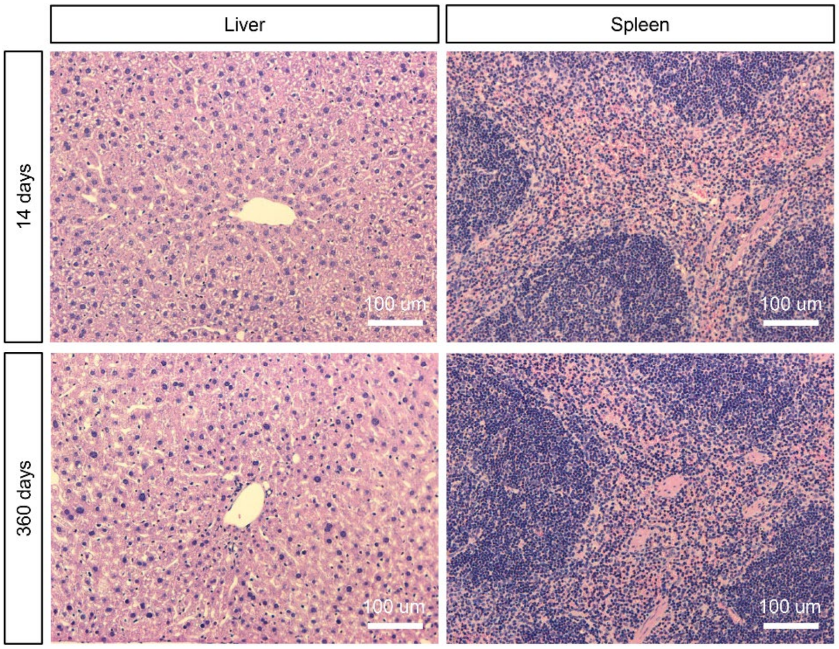

Supplemental Figure 3. Assessment of liver and spleen histology in AAV2-GEC transduced mice

H&E staining in livers and spleens from AAV2-GEC transduced C57BL/6J mice at 14 and 360 days after intravenously injection. N=3 per time point. No obvious histological lesions were observed.

Supplemental figure 4

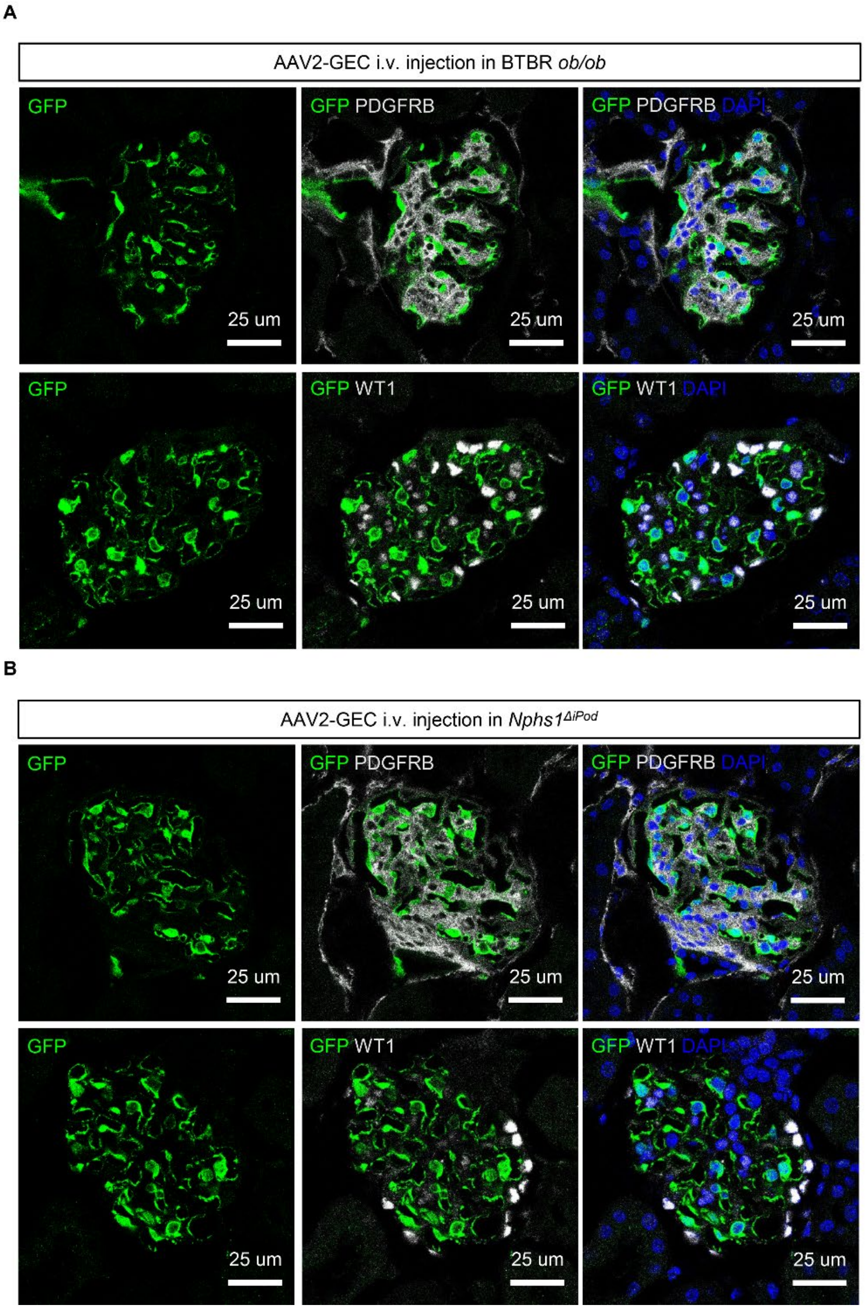

55 ***Supplemental Figure 4. AAV2-GEC-mediated GFP expression in the glomeruli of GFB-damaged mice***
Representative overview images of AAV2-GEC-mediated GFP expression in kidneys from (**A**) BTBR *ob/ob* and (**B**) *Nphs1*$^{\Delta iPod}$ mice. AAV2-GEC-mediated GFP expression was not detected in mesangial cells marked by anti-PDGFRB antibody or podocytes marked by anti-WT1 antibody. Nuclei were counterstained with DAPI.

Supplemental figure 5

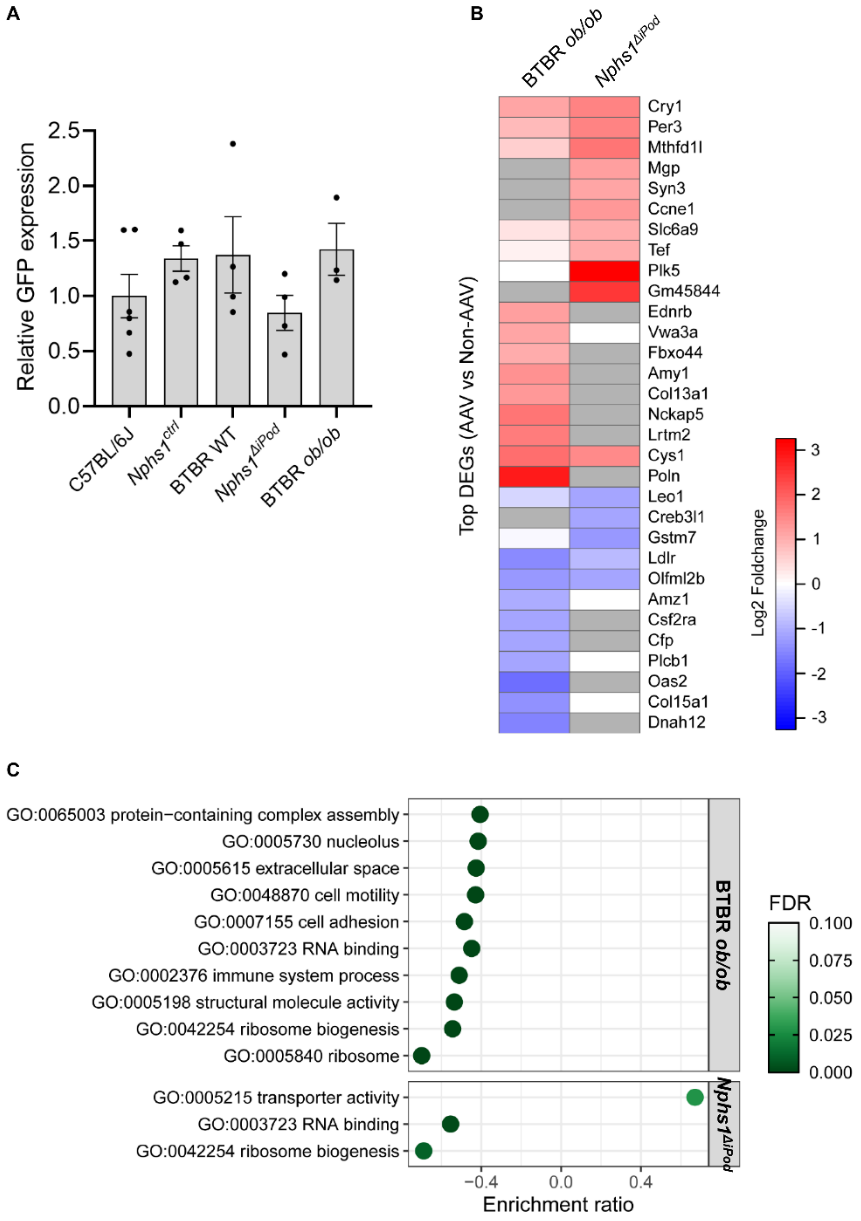

Supplemental Figure 5. Transduction efficiency of AAV2-GEC in healthy and disease conditions

(**A**) Relative expression of *GFP* in GEC isolated from C57BL/6J, *Nphs1*^{ctrl}, BTBR WT, *Nphs1*$^{\Delta iPod}$ and BTBR *ob/ob* mice. Values are mean ± SEM. (**B**) Heatmap showing the significant DEGs identified in AAV2-GEC injected GEC versus non-injected GEC from male BTBR *ob/ob* and *Nphs1*$^{\Delta iPod}$ mice (n=3 in each condition). Nonsignificant genes are shown in grey. Significance: log2FC ≥1 or ≤-1, FDR≤0.05. (**C**) Dot plot showing significant GO in BTBR *ob/ob* and *Nphs1*$^{\Delta iPod}$. Significance: FDR≤0.05. DEG: differentially expressed genes, FC: fold change, FDR: false discovery rate, GO: gene ontology.

Supplemental figure 6

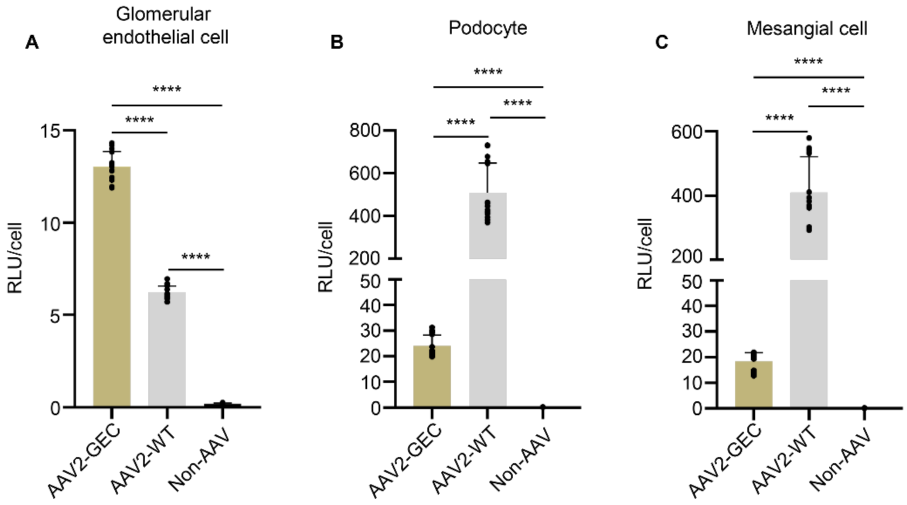

Supplemental Figure 6. Targeting effect of AAV2-GEC in human glomerular cell types

Transduction of (**A**) human primary GEC, (**B**) immortalized human podocytes and (**C**) human primary mesangial cells at a MOI of 1E5 viral genomes per cell with AAV2-GEC or AAV2-WT carrying a luciferase reporter gene. Cells without transduction were used as mock controls (Non-AAV). Luciferase activities were measured 24 hours after transduction and are given in RLU per cell. N=18 from 3 independent experiments per condition. Values are mean + SD. Significance: one-way ANOVA with Dunnett's test, ****P < 0.0001.

Supplemental figure 7

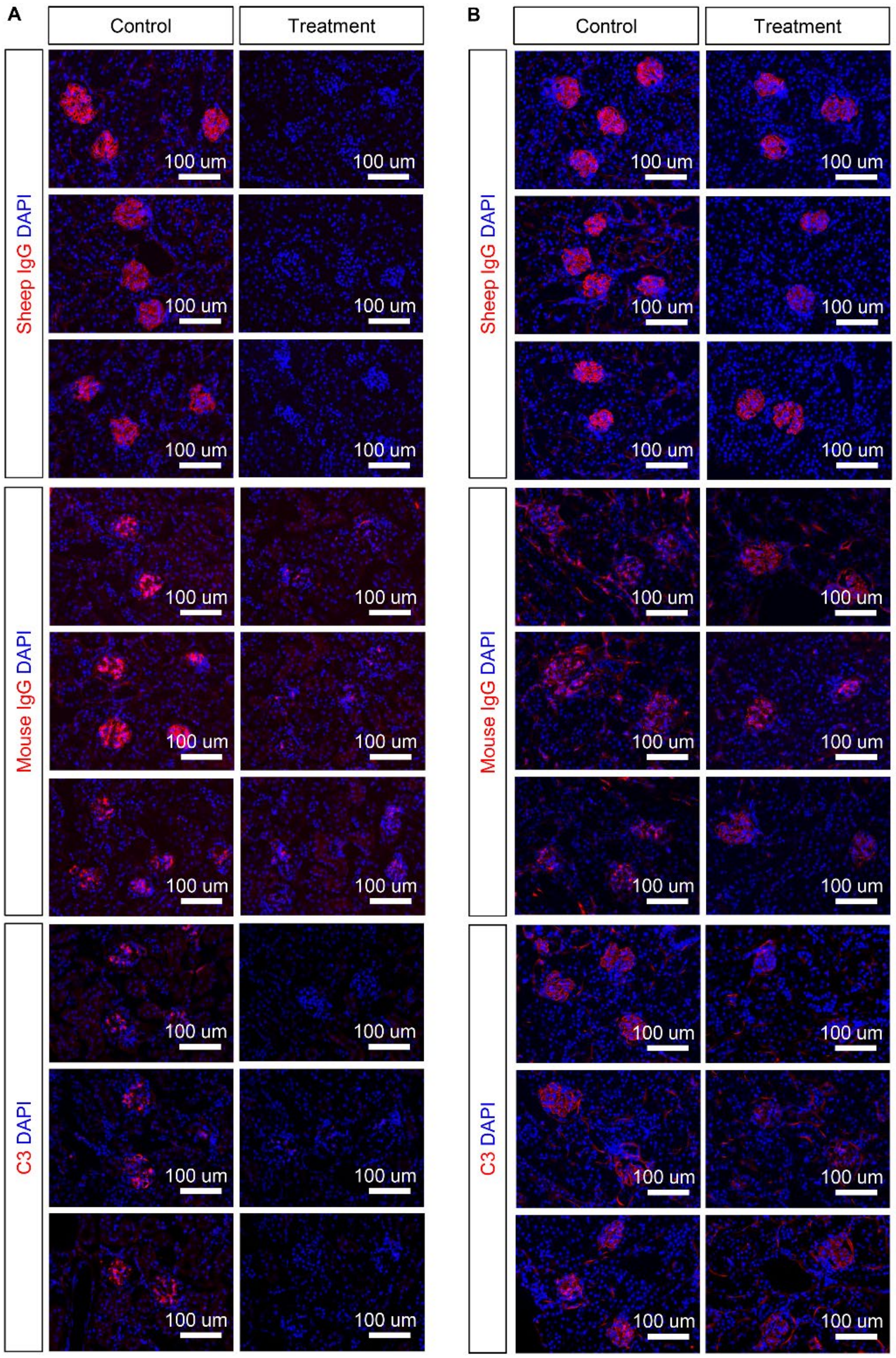

***Supplemental Figure 7*. Immunoglobulin and complement deposition in the kidney**

Representative images showing the deposition of sheep IgG, mouse IgG and C3 in the kidneys (**A**) at 7 days of prophylactic intervention and (**B**) at 22 days of therapeutic intervention. Nuclei were counterstained with DAPI.

Supplemental figure 8

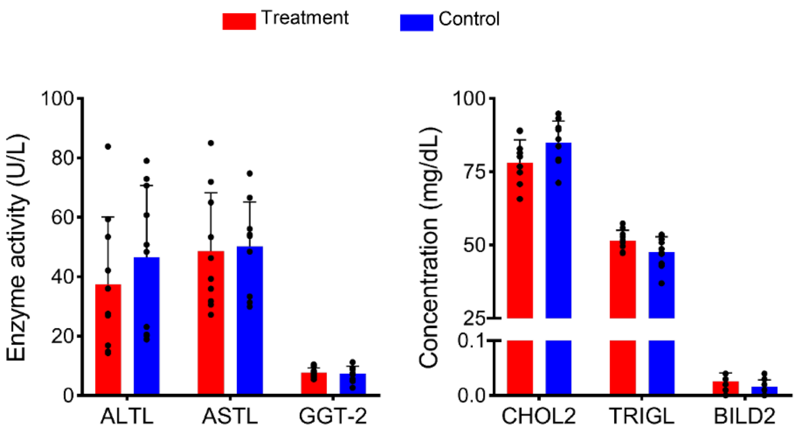

Supplemental Figure 8. Assessment of liver functions in AAV2-GEC-IdeS treated mice

Liver functions were evaluated in mice from treatment (AAV2-GEC-IdeS) and control (AAV2-GEC-GFP) groups. No statistically significant differences were found. Values are mean + SD.

Supplemental figure 9

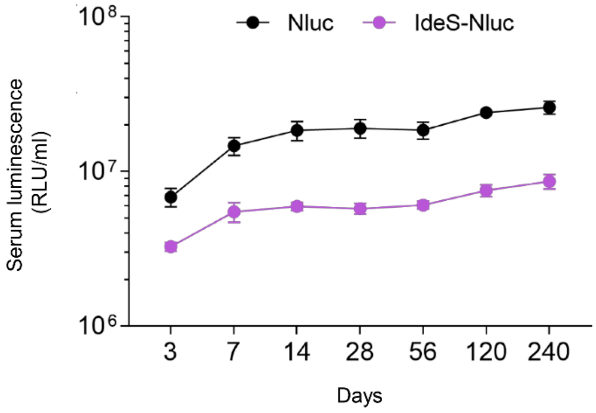

95 ***Supplemental Figure 9. Serum IdeS concentration in AAV2-GEC-IdeS mice***

C57BL/6J mice were injected intravenously with AAV2-GEC-IdeS-Nluc (shown as IdeS-Nluc) and AAV2-GEC-Nluc (shown as Nluc) as control. The serum IdeS concentration was measured by the luminescence at 3, 7, 14, 28, 56, 120 and 240 days after injection. N≥7 per each group. Values are mean ± SEM. Nluc: Nanoluciferase.

Supplemental Methods

Microscope and long-term GFP signal intensity quantification

Two AAV-GEC-GFP injected C57BL/6J mice for each time point and two wild-type C57BL/6J mice were sacrificed. Both kidneys were harvested, fixed and embedded in OCT. Seven um cryo-sections from all samples were stained with chicken anti-GFP (1:500, Thermo Scientific, A10262) together. The exposure setting was adjusted according to the highest signal intensity at 14-day and maintained for all samples. Ten images from each kidney (incl. 20-30 glomeruli) were taken by Zeiss Apotome with 20x/0.8 objective. Each glomerulus in image was selected with polygon in ImageJ, and the mean intensity (RawIntDen/Aera) was used for quantification. The final data were presented as relative expressions, i.e., each time point was subtracted from the wild-type and then compared to the 14-day data.

Transduction of human glomerular cell types

Human primary glomerular endothelial cells (Cell Biologics, H-6014G) were cultured in 24-well plates coated with 0.1% gelatin solution (Millipore, ES-006-B) in Complete Human Endothelial Cell Medium (Cell Biologics, H1168). Human primary renal mesangial cells (ScienCell, 4200) were cultured in 24-well plates coated with poly-L-Lysine (2ug/cm2) in MsCM (ScienCell, 4201). Immortalized human podocytes (provided by M. Saleem, University of Bristol, UK) were cultured and differentiated in 24-well plates in the RPM1 1640 medium (Sigma Aldrich, R7388) supplemented with 10% fetal bovine albumin (FBS), 1% Penicillin/Streptavidin (P/S), Moin supplement (1M HEPES, 50 mM Sodium pyruvate and minimum essential medium (MEM)) and 0.1% Insulin Selenite Transferrin (ITS). The three human glomerular cell types were transduced with AAV2-GEC or AAV2-WT carrying a secreted nanoluciferase (supplemental methods) at the multiplicity of infection (MOI) 1E5 viral genomes per cell. 24 hours after transduction, the transduction efficiency was measured by the luciferase activity in the medium. The luciferase activity was given in relative light units (RLU) per cell.

MACS-sorted glomerular endothelial cells

GEC were isolated from 3 male mice for each condition. The protocol for isolation of glomeruli from mouse kidneys and digestion of glomeruli was modified from a previous publication (1). Briefly, glomeruli were isolated using the magnetic bead method, and the isolated glomeruli were resuspended in 2 ml digestion solution (Roche, 1000 U/ml Liberase TL [5401020001], and 50 U/ml DNase I [Roche, 4536282001] in RPMI 1640) and incubated at 37 °C for 60 min on a thermomixer shaker at 1,400 rpm. During incubation, different mechanical forces were applied to dislodge the cells. Immediately thereafter, the beads were removed with a magnet (DynaMag™-2 Magnet) rack and ten times the volume of cold HBSS supplemented with 10% FBS was added to the single cell suspension. The cell suspension was sieved through a 30 µm cell strainer and centrifuged at 400 ×*g* for 4 min at 4 °C. The cell pellet was resuspended in staining buffer

(Miltenyi, 130-091-376), and stained with CD31 microbeads (Miltenyi, 130-097-418) at 4 °C for 15 min. After staining, CD31-positive cells were selected using a LS MACS column (Miltenyi, 130-042-201) according to the manufacturer's protocol. A total of 100,000-300,000 cells were collected for RNA isolation. RNA was isolated using an RNeasy Micro Kit (Qiagen, 74004) according to the manufacturer's protocol.

Bulk RNA-sequencing and data analysis

All samples were sent to BGI TECH SOLUTIONS (Hong Kong, China). Libraries were prepared using the Stranded mRNA (transcriptome) library preparation protocol and sequenced on the DNBseq platform as paired end runs (150 bases) with 20 million reads per sample.

Sequence reads were processed with fastp (v0.20.1) to remove sequences originating from sequencing adapters and sequences of low quality using the program's default parameters (2). Reads were then aligned to the mouse reference assembly (GRCm39.110) using STAR (v2.7.9a) (3). Differential expression was assessed using DESeq2 (v1.42.0) (4). Only protein-coding genes were investigated. A gene was considered significantly differentially expressed if the corresponding absolute log2-transformed fold change (log2FC) was not less than 1 and, in addition, the false discovery rate (FDR) did not exceed a value of 0.05. Gene set enrichment analysis (GSEA) was performed with clusterProfiler (v3.14) (5) in combination with a selection of the Molecular Signatures Database (MSigDB) subsets C2 and H. In addition to MSigDB, the Gene Ontology (GO) resource (6) was employed in combination with clusterProfiler.

Real-time PCR

For the evaluation of the AAV2-GEC transduction efficiency in healthy and diseased states, AAV2-GEC-GFP was intravenously injected into different mice at a dose of 5E12 vg/kg. 14 days after AAV injection, the GEC were isolated using the MACS-sorted method (mentioned above). Total RNA was extracted using the RNeasy Micro Kit (Qiagen, 74004) following the manufacturer's protocols. Subsequently, cDNA was synthesized from total RNA using the ProtoScript® II First Strand cDNA Synthesis Kit (New England Biolabs, E6560L) in the presence of Oligo $d(T)_{23}$ VN for all samples. Transgene expression was quantified using the QuantStudio 3 Real-Time PCR System (Thermo Scientific) with the following primers: GFP-specific primers: 5'-CTACGGCGTGCAGTGCTTCAG-3' (forward), 5'-CTTCAGCTCGATGCGGTTCAC-3' (reverse). The relative expression level of GFP was obtained by normalization to the total RNA.

Histology

Mouse was perfused and the tissues were fixed as described above. For hematoxylin and eosin staining, specimens from liver and spleen were embedded in paraffin blocks. Sections were stained with H&E according to standard procedures and evaluated under a light microscope.

Measurements of the kidney and liver functions

Urinary albumin was measured using a mouse albumin ELISA quantitation kit (Bethyl, E90-134). Urinary creatinine was measured using Creatinine Jaffé Gen. 2 kit (Roche, 04810716190) according to the manufacturer´s recommendation. Liver functional index levels were measured using kits from Roche according to the instruction.

Plasmid construction

The IdeS gene (mentioned above) with the secreted signal peptide of albumin and fusion to the nanoluciferase (Nluc) gene (sequence from pNL1.1[Nluc], Promega) was synthesized in ThermoFisher Scientific. The synthesized fragment was subcloned into the vector pscAAV-CMV-GFP (Addgene, plasmid 32396) by replacing the GFP with AgeI and BsrGI to generate the pscAAV-CMV-IdeS-Nluc vector. Similarly, The Nluc gene with the secreted signal peptide of albumin was synthesized and subcloned to generate pscAAV-CMV-Nluc vector.

Assessment of Nanoluciferase (Nluc) activity in serum

C57BL/6J mice were injected intravenously with AAV2-GEC-IdeS-Nluc and AAV2-GEC-Nluc. On day 3, 7, 14, 28, 56, 120 and 240 after injection, mice were anesthetized with isoflurane and blood was collected through the submandibular vein. Serum was diluted at a ratio of 1:10. 100 ul of diluted serum from each sample was then added to an opaque 96-well microtiter plate and one volume of Nano-Glo® Luciferase Assay Reagent equal to the sample volume was added to the same well. After 5 minutes of incubation, the luminescence of the samples was measured using a luminometer (Berthold technologies, Mithras LB 940).

References

1. Liu S, Zhao Y, Lu S, Zhang T, Lindenmeyer MT, Nair V, et al. Single-cell transcriptomics reveals a mechanosensitive injury signaling pathway in early diabetic nephropathy. *Genome Med.* 2023;15(1):2.
2. Chen S, Zhou Y, Chen Y, and Gu J. fastp: an ultra-fast all-in-one FASTQ preprocessor. *Bioinformatics.* 2018;34(17):i884-i90.
3. Dobin A, Davis CA, Schlesinger F, Drenkow J, Zaleski C, Jha S, et al. STAR: ultrafast universal RNA-seq aligner. *Bioinformatics.* 2013;29(1):15-21.
4. Love MI, Huber W, and Anders S. Moderated estimation of fold change and dispersion for RNA-seq data with DESeq2. *Genome Biol.* 2014;15(12):550.
5. Wu T, Hu E, Xu S, Chen M, Guo P, Dai Z, et al. clusterProfiler 4.0: A universal enrichment tool for interpreting omics data. *Innovation (Camb).* 2021;2(3):100141.
6. Ashburner M, Ball CA, Blake JA, Botstein D, Butler H, Cherry JM, et al. Gene ontology: tool for the unification of biology. The Gene Ontology Consortium. *Nat Genet.* 2000;25(1):25-9.
